# A Meta-Analysis of Clinical and Echocardiographic Outcomes of Physiological Versus Conventional Pacing

**DOI:** 10.3390/biomedicines13061359

**Published:** 2025-05-31

**Authors:** Patrycja Paluszkiewicz, Adrian Martuszewski, Jacek Smereka, Jacek Gajek

**Affiliations:** 1 Department of Emergency Medical Service, Wroclaw Medical University, ul. Parkowa 34, 51-616 Wrocław, Poland; 2 Division of Environmental Health and Occupational Medicine, Department of Population Health, Wroclaw Medical University, Mikulicza-Radeckiego 7, 50-368 Wrocław, Poland; 3 Medical Faculty, Wrocław University of Science and Technology, 50-368 Wrocław, Poland

**Keywords:** His bundle pacing, left bundle branch area pacing, left bundle branch pacing, heart failure, biventricular pacing, right ventricular pacing, cardiac resynchronization therapy, atrial fibrillation, sinus node disease

## Abstract

**Background**: Conduction system pacing (CSP), encompassing His bundle *pacing* (HBP) and left bundle branch area pacing (LBBAP), has emerged as an alternative to conventional pacing methods such as right ventricular pacing (RVP) and biventricular pacing (BVP). This meta-analysis aimed to compare the effects of CSP versus conventional pacing on left ventricular function and selected clinical and electrophysiological outcomes. **Methods**: Prospective and retrospective studies (randomized, observational, registry-based) reporting pre-post data or direct comparisons between CSP (HBP, LBBAP) and conventional methods (BVP, RVP) for at least one of LVEF, LVESV, LVEDV, QRS duration, NYHA class, NT-proBNP, R-wave, or pacing threshold were included. PubMed and Web of Science databases were searched up to 31 March 2025. Quality assessment (QualSyst), publication bias (Egger’s test, trim-and-fill), subgroup analyses, and meta-regression (follow-up duration) were performed. The review was registered in the INPLASY database (INPLASY202540050). **Results**: 28 studies (8777 patients, 47 comparisons) were included. CSP significantly improved LVEF (*SMD* = 1.16; 95%CI: 0.94–1.38), shortened QRS duration (SMD = 0.75; 95%CI: 0.24–1.26), and reduced NYHA class (SMD = 1.94; 95%CI: 1.59–2.29), NT-proBNP levels (SMD = 1.27; 95%CI:0.85–1.69), LVEDV (SMD = 0.90; 95%CI: 0.42–1.38), and LVESV (SMD = 1.31; 95%CI: 0.81–1.81). In head-to-head comparisons, LBBAP and HBP showed similar efficacy, both superior to conventional pacing. Improvement in LVEF significantly correlated with longer follow-up (*p* = 0.004). Publication bias was non-significant (Egger *p* = 0.15), despite high heterogeneity (I^2^ > 90%). **Conclusions**: CSP demonstrated superior clinical and echocardiographic outcomes compared to conventional pacing. Limitations include the predominance of non-randomized studies, high heterogeneity, and variability in follow-up duration, supporting the need for high-quality randomized trials.

## 1. Introduction

Cardiac pacing is a widely used method for the treatment of heart rhythm and conduction disorders. In ESC member countries, pacemaker implantation rates range from fewer than 60 to more than 1000 procedures per million inhabitants, reflecting the growing frequency of these interventions in an aging population [1]. Conventional right ventricular pacing (RVP) can effectively prevent bradycardia; however, its mode of cardiac activation is non-physiological. Delivery of the electrical impulse directly into the right ventricular myocardium leads to electrical and mechanical dyssynchrony of the ventricles, as activation propagates outside the natural conduction system. Chronic RVP may impair left ventricular function and promote the development of heart failure (HF), pacemaker-induced cardiomyopathy (PICM), and arrhythmias, particularly atrial fibrillation (AF) [2]. The PACE study demonstrated that patients undergoing RVP experienced adverse left ventricular remodeling, deterioration of systolic function, and more frequent heart failure hospitalizations (HFHs) compared to patients with conventional biventricular pacing (BVP), even despite a relatively preserved ejection fraction [3]. To limit the adverse dyssynchrony induced by RVP, the technique of BVP was developed, which forms the basis of cardiac resynchronization therapy (CRT). It involves simultaneous stimulation of the left and right ventricles, typically by means of a lead positioned in a coronary vein of the left ventricle and a lead in the right ventricle. BVP improves cardiac function and exercise capacity, reverses adverse remodeling, and reduces HFH and mortality in patients with HF and wide QRS. It has proven superior to RVP across different NYHA classes [4,5,6,7,8]. Despite these benefits, approximately 30% of patients do not experience improvement after conventional CRT and are classified as CRT non-responders [9,10]. The causes of non-response to CRT are multifactorial. They may include unfavorable anatomy of the coronary venous system hindering optimal placement of the left ventricular lead, phrenic nerve stimulation, extensive scarring in the left ventricular myocardium, or inappropriate patient selection [11]. The above limitations of conventional pacing highlight a significant issue of suboptimal ventricular contraction synchronization in a subset of patients, particularly those who do not benefit from CRT.

In recent years, significant progress has been made in the field of cardiac electrotherapy, focusing on physiological myocardial activation. The most important achievement is the development of the concept of conduction system pacing (CSP). This technique involves placing the lead directly within the cardiac conduction system, allowing for ventricular activation in a manner closely resembling the natural one. CSP primarily includes His bundle pacing (HBP) and left bundle branch area pacing (LBBAP). The first successful implantation of a lead for permanent HBP was described in 2000, marking the beginning of a new era in cardiac pacing [12,13,14]. Initially, HBP faced technical limitations related to lead positioning and high thresholds, overcome by the development of dedicated tools and better anatomical understanding [13,15]. A more recent method of CSP is LBBAP [16]. In this technique, the lead is advanced deeply into the interventricular septum to capture the fibers of the left bundle branch of the conduction system. In clinical practice, LBBAP has proven to be easier and more effective to implant than HBP. It is characterized by a higher success rate, lower pacing thresholds, and better signal parameters, while simultaneously presenting a lower risk of lead dislodgement [17].

A key advantage of CSP techniques is the direct activation of the His–Purkinje network, which enables synchronous contraction of both ventricles, thereby bringing the cardiac mechanics closer to physiological conditions [14,18,19]. Clinical studies have shown that CSP results in a narrower QRS complex, better contraction synchrony, and a higher left ventricular ejection fraction (LVEF) compared with conventional pacing. In patients undergoing HBP and LBBAP, there is no decline in ejection fraction observed with long-term RVP. QRS widening is significantly less pronounced than during RVP. Moreover, in patients requiring CRT, CSP can provide an improvement in left ventricular function at least comparable to that achieved with conventional BVP, while offering a lower risk of arrhythmias and other complications [20,21,22]. Physiological cardiac pacing may favorably influence the course of HF, particularly in patients with pre-existing ventricular contraction dyssynchrony.

Despite the promising results of numerous studies, there remains a knowledge gap regarding the actual superiority of physiological pacing over conventional techniques. The studies published to date on HBP and LBBAP are often observational in nature, involve relatively small patient cohorts, and use variously defined endpoints, which complicates the clear interpretation of their results. Moreover, there is no consensus on which CSP technique is optimal. Direct comparisons between HBP and LBBAP indicate similar clinical efficacy of both approaches. In comparative analyses, improvements in ejection fraction and QRS narrowing achieved with LBBAP were found to be comparable to those obtained with HBP, with both approaches outperforming conventional BVP [23]. Moreover, the lack of large, randomized clinical trials confirming preliminary observations has prevented physiological pacing from being widely incorporated into clinical guidelines and routine practice [24].

Given the scattered data and the lack of large randomized studies, we aimed to provide a comprehensive synthesis of current evidence through this meta-analysis. Table 1 presents the PICO framework, outlining the study population, intervention, comparator, and outcome measures. By addressing this issue, we seek to support the optimization of device-based therapy in patients requiring chronic cardiac pacing.

## 2. Materials and Methods

The systematic review was conducted in accordance with the PRISMA 2020 guidelines [25,26]. This review also followed the PRISMA-NMA guidelines as applicable to pairwise meta-analyses with some indirect comparisons, but did not apply full NMA modeling due to limited connectedness of evidence. The study protocol was registered in the INPLASY database (ID: INPLASY202540050). No changes were made to the original protocol after its registration. The framework of the review was defined according to the PICO format, as outlined in the Introduction.

Original studies reporting quantitative pre-post data or head-to-head comparisons for the following pacing techniques were included in the analysis: HBP, LBBAP, BVP, and RVP. Studies were eligible if they reported at least one of the primary endpoints: LVEF, LVEDV, LVESV, QRS duration, NYHA class, NT-proBNP levels, R-wave amplitude, or pacing threshold. Conference abstracts, review articles, case reports, publications without full text, and studies lacking data necessary to calculate the standardized mean difference (SMD) were excluded. A systematic search was conducted in the PubMed and Web of Science databases for articles published up to 31 March 2025. Literature searches were performed independently by two reviewers, each conducting two rounds of screening in both PubMed and Web of Science. Advanced search strategies were used with the following query: (“his bundle pacing”) OR (“left bundle branch pacing”) OR (“left bundle branch area pacing”) OR (“HOT-CRT”) OR (“LOT-CRT”). The search results were limited to studies published between 2014 and 2025 and to the following types of publications: Full Text, Classical Article, Clinical Study, Clinical Trial (all phrases), Clinical Trial Protocol, Comparative Study, Controlled Clinical Trial, Multicenter Study, Observational Study, Randomized Controlled Trial.

Data selection and extraction were performed independently by two authors (A.M. and P.P.). Discrepancies were resolved by consensus. Data were extracted into a standardized spreadsheet that included the study name, year of publication, type of pacing, group sizes, mean values, standard deviations before and after the intervention, and follow-up duration. In cases of incomplete data, attempts were made to contact the study authors. The primary endpoints included LVEF, LVEDV, LVESV, QRS duration, NYHA class, NT-proBNP levels, R-wave amplitude, and pacing threshold. Additionally, data were collected on patient age, the etiology of HF, and the length of follow-up. NT-proBNP concentrations were analyzed on a linear scale; log10 transformation was not possible due to the lack of access to raw data. Studies were assigned to comparative analyses based on the type of pacing declared by the original authors for each study arm. Data were grouped for analyses such as CSP versus conventional pacing, HBP versus BVP, LBBAP versus RVP, and others, depending on the availability of numerical data. Only multi-arm studies were included in the head-to-head analyses. Results were presented as forest plots, organized by type of intervention and year of publication.

Methodological quality was assessed using the QualSyst tool for quantitative studies [27]. Two authors (A.M. and P.P.) independently assessed the studies, assigning scores from 0 to 2 across 14 criteria. The final score was presented as the percentage of the maximum possible points. The risk of publication bias was evaluated using Egger’s test and the trim-and-fill method. For each study, the final score was calculated according to the following formula:

$$Summary\;QualSyst\;score\;=\;\frac{\left(number\;of\;“yes”\times 2\right)+\left(number\;of\;“partials”\times 1\right)}{28-\left(number\;of\;“N/A”\times 2\right)}$$

where

- “yes” indicates the number of positive responses fully meeting a given criterion (2 points),
- “partials” indicates the number of responses partially meeting a given criterion (1 point),
- “N/A” indicates questions deemed not applicable to a given study and excluded from the denominator.

The final QualSyst score was presented as a percentage value reflecting the overall study quality. Two reviewers independently assessed each study, and any discrepancies were resolved by consensus.

Effects were expressed as SMD with 95% Confidence Intervals (CIs). Based on the data extracted from the publications, pooled standard deviations (s pooled), SMD with 95%CI, and Standard Errors (SEs) were calculated in accordance with Cochrane guidelines [28]. No efficacy rankings or SUCRA (Surface Under the Cumulative Ranking Curve) analyses were performed, as no formal network meta-analysis (NMA) was conducted and no ranking model was constructed. A random-effects model with REML estimation was applied for the analyses. General analyses, between-group comparisons, subgroup analyses, meta-regressions (with follow-up duration as a moderating variable), and head-to-head comparisons were conducted. Heterogeneity was assessed using the I^2^ and τ^2^ statistics. Tests for asymmetry were not performed when the number of comparisons was fewer than 10. A formal NMA was not conducted because the available data did not allow for the construction of a coherent comparison network. In many studies, multiple pacing techniques were analyzed without a common comparator, and for several endpoints, complete numerical data were missing. Attempts to build an NMA model in the R environment were unsuccessful due to the structure of the data. All analyses were performed using RStudio (version 2024.12.0 Build 467) with the meta, metafor, dmetar, dplyr, readxl, and grid packages.

Sensitivity analyses for potential file drawer bias were conducted separately for each key endpoint (LVEF, NYHA class, LVESV, LVEDV). Rosenthal’s Fail Safe N was calculated using the fsn() function from the metafor package (R version 4.3.2, REML method). Results are presented as SMD. The ‘favorable’ direction for each variable is summarized in Table 2. For outcomes where a lower value is clinically desirable (e.g., NYHA class, NT-proBNP levels), the SMD is expressed as a positive value by inverting the sign during calculations.

Forest plots present the results of SMD analyses for selected endpoints, including 95%CI. On the left side of the plot, the names of the compared groups and source data (author, year) are shown, while on the right side, the SMD value with its corresponding 95%CI and the study’s weight in the analysis are displayed. Each square represents the point estimate of an individual study, and its width reflects precision (weight in the random-effects model). The horizontal line through the center of the square represents the CI, while the final diamond symbolizes the pooled effect for all studies in a given analysis. The horizontal X-axis shows the range of SMD, with 0 indicating no difference between groups. The vertical solid line at SMD = 0 serves as a reference point—if the CI crosses this line, the result is not statistically significant. The solid vertical line represents no difference between groups (SMD = 0), while the dashed line indicates the pooled effect estimated by the random-effects model. In some plots, different graphical symbols (e.g., grey squares with lines, diamonds) were used, following the same schematic meaning.

Bubble plots present meta-regression analyses evaluating the impact of follow-up duration (in months) on the magnitude of the intervention effect expressed as the SMD. Each point on the plot represents a single study, with its position determined by the follow-up length (X-axis) and the estimated effect size (Y-axis). The solid red line represents the regression line, showing the trend between follow-up duration and effect size. The dashed red lines indicate the 95%CI for this regression. A positive slope of the line suggests that a longer follow-up duration may be associated with greater improvement in the analyzed clinical parameter. The size of the points (bubbles) is uniform in this plot—each point has the same size, meaning that no weighting by precision was applied (unless stated otherwise in the figure caption).

The certainty of the results was assessed using the GRADE scale and indirectly based on the consistency of effects, the number of analyses, the distribution of values, and the results of publication bias assessment [29]. The source code (R script), data extraction templates, and input data files are available upon request from the corresponding author. The data are not publicly available due to restrictions stemming from the original publications.

## 3. Results

**Figure 1:**
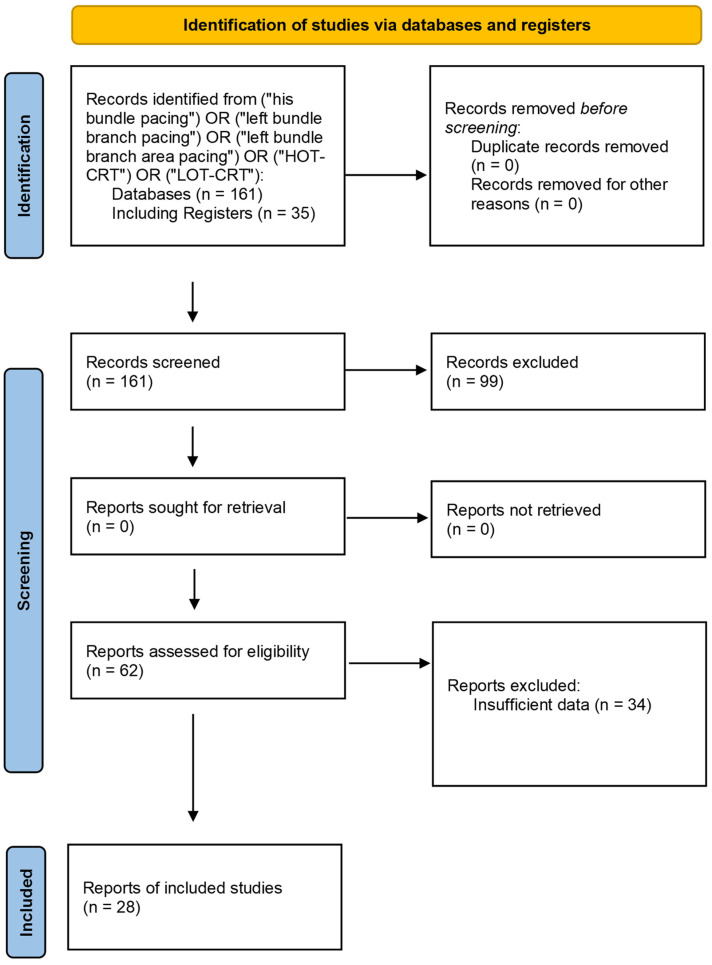
The flow diagram illustrates the flow diagram for material preparation and the methodological framework of this study [26].

A total of 161 publications were identified through the search process. After screening titles and abstracts, 99 studies were excluded due to failure to meet the inclusion criteria—most commonly because of a lack of comparable data for key endpoints, analyses of populations outside the predefined criteria (e.g., pediatric studies, patients after transplantation), incomplete numerical information, or an inappropriate type of publication (e.g., reviews, commentaries, single case reports). Among the 62 articles assessed in full text, additional studies were excluded due to the absence of complete numerical data or lack of access to the full text. Ultimately, 28 studies comprising a total of 8777 patients and 47 comparisons were included in the final analysis. Most of the analyses were based on pre-post comparisons (*n* = 46), while one comparison involved the direct assessment of RVP against another pacing technique. Figure 1 presents the flow diagram and methodological framework of the present review.

Both randomized and observational studies, including prospective studies, retrospective studies, and registry-based analyses, were included in the meta-analysis. The comparisons evaluated HBP, LBBAP, BVP, and RVP. The follow-up duration across studies ranged from 3.9 to 60 months, with a median follow-up of 12 months. The data encompassed pre- and post-implantation values as well as direct comparisons between CSP and conventional pacing groups, assessing parameters such as LVEF, QRS duration, NYHA class, NT-proBNP levels, LVEDV, LVESV, and lead characteristics: R-wave amplitude and pacing threshold. Across the included studies, patients were predominantly indicated for pacing due to either bradyarrhythmias (e.g., atrioventricular block, sick sinus syndrome) or heart failure requiring CRT. When reported, baseline characteristics commonly included reduced or mildly reduced LVEF, NYHA class II–III symptoms, and prolonged QRS duration. A detailed summary of clinical features is now provided in the updated Table 3.

Methodological quality assessments were conducted using the QualSyst tool for evaluating quantitative studies. Each study was assessed across 14 methodological categories, with a scoring system ranging from 0 to 2 (where “2” indicated full compliance with the criterion, “1” partial compliance, and “0” non-compliance). When a criterion was not applicable, it was marked as “N/A” and excluded from the calculation of the final score. The overall score was expressed as the percentage of possible points after adjusting for “N/A” items. The mean methodological quality score for the 28 included studies was 0.77 (range: 0.68 to 1.00). Three studies achieved a score of ≥0.85 and were classified as high methodological quality [46,50,52]. Most studies (*n* = 25) scored between 0.71 and 0.79 and were rated as moderate quality, while one study [39] scored below 0.70, classifying it as low methodological quality. The most common methodological limitations included a lack of blinding of outcome assessors, incomplete reporting of randomization procedures, and insufficient description of data collection methods. Nevertheless, most studies provided comprehensive information in key areas such as endpoint definitions, intervention descriptions, and statistical analysis methods.

According to the GRADE [29] assessment (Table 4), the certainty of evidence was low for all analyzed endpoints (including LVEF and NYHA), primarily due to the lack of randomized studies and very high inconsistency (I^2^ > 90%).

### 3.1. Effects on Left Ventricular Ejection Fraction (LVEF)

In the meta-analysis including 34 comparisons derived from 20 unique publications, the cumulative effect of various cardiac pacing techniques on LVEF was estimated. The analysis demonstrated a clear and statistically significant improvement in LVEF following lead implantation; the pooled SMD was −1.38 (95%CI: −1.69 to −1.06; *p* < 0.0001). Heterogeneity among the studies was very high (I^2^ = 94.5%), which justified the performance of stratified analyses. Figure S1 presents the distribution of individual study effects along with the cumulative effect in the random-effects model.

**Figure 2:**
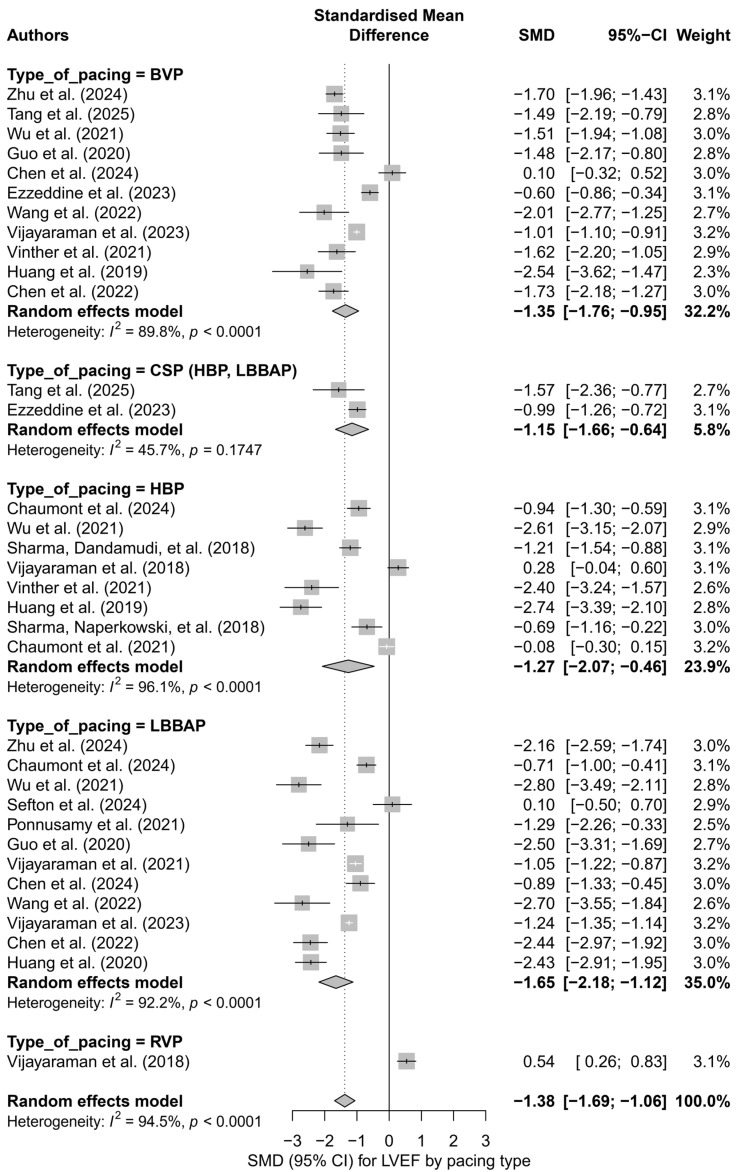
Forest plot for the subgroup analysis according to the type of pacing. Subgroups: HBP, LBBAP, CSP (HBP + LBBAP), BVP, RVP. The greatest improvement in LVEF was observed in the LBBAP group (SMD = −1.65), while the smallest effect was noted in the RVP group (SMD = 0.54). The differences between groups were statistically significant (*p* < 0.0001). Heterogeneity was elevated in all subgroups except for CSP (I^2^ = 45.7%) [21,30,31,32,34,35,36,37,38,39,41,43,44,45,48,49,50,51,53,55].

To identify potential sources of this variability, a subgroup analysis was conducted based on the type of pacing technique. The greatest improvement in LVEF was observed for LBBAP (SMD = −1.65; I^2^ = 92.2%) and HBP (SMD = −1.27; I^2^ = 96.1%). A significant effect was also noted for BVP (SMD = −1.35), whereas RVP showed a weak, positive effect (SMD = 0.54), suggesting no improvement or a possible deterioration of left ventricular function. The differences between subgroups were statistically significant (*p* < 0.0001). Figure 2 presents a detailed subgroup comparison, illustrating SMD values, and CI and heterogeneity levels.

**Figure 3:**
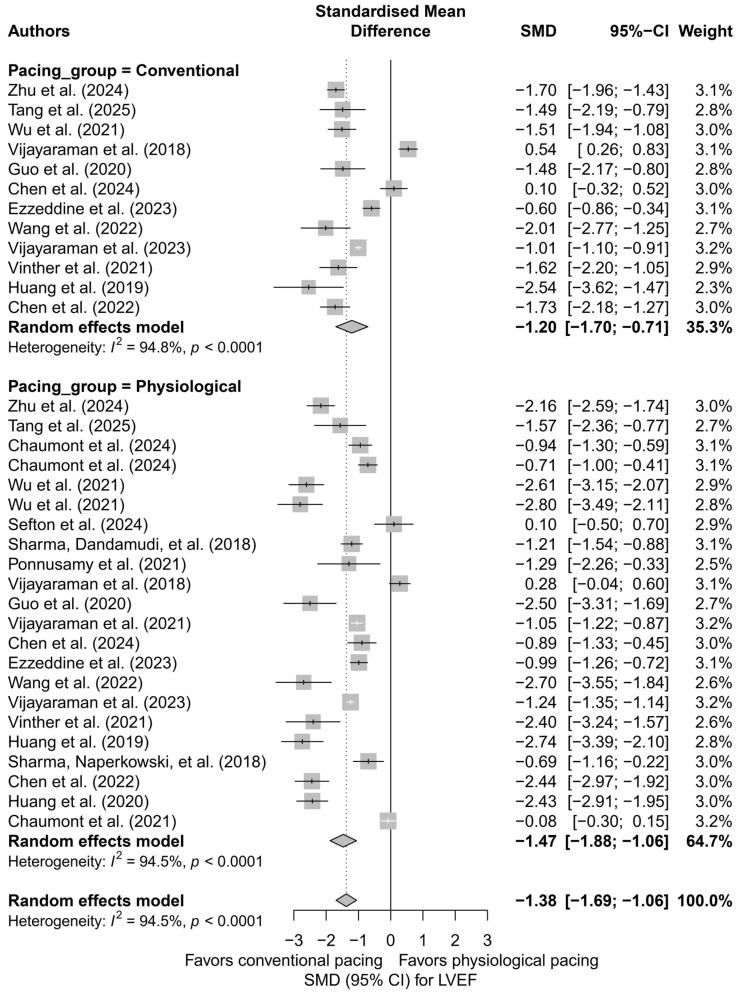
Forest plot for the comparison of physiological pacing (HBP, LBBAP, CSP) versus conventional pacing (BVP, RVP)—effect on LVEF (SMD). The difference between groups was not statistically significant (*p* = 0.416), but a trend toward a stronger effect in the physiological group was evident [21,30,31,32,34,35,36,37,38,39,41,43,44,45,48,49,50,51,53,55].

Subsequently, physiological techniques (LBBAP, HBP) were compared with conventional techniques (BVP, RVP). An improvement in LVEF was observed in both groups, but it was markedly stronger in the physiological group (SMD = −1.47; 95%CI: −1.88 to −1.06) compared to the conventional group (SMD = −1.20; 95%CI: −1.70 to −0.71). The difference between these classes did not reach statistical significance (*p* = 0.416), but a trend favoring physiological pacing was noted. Figure 3 illustrates this division, presenting the data graphically along with the heterogeneity within both groups.

In the head-to-head analysis, which included four studies directly comparing HBP and LBBAP, both techniques demonstrated a comparable and significant improvement in LVEF. For HBP, the SMD was −1.76 (95%CI: −3.40 to −0.13), and for LBBAP, it was −1.73 (95%CI: −3.79 to 0.33). The difference between these techniques was not statistically significant (*p* = 0.98), indicating their equivalence in this regard.

**Figure 4:**
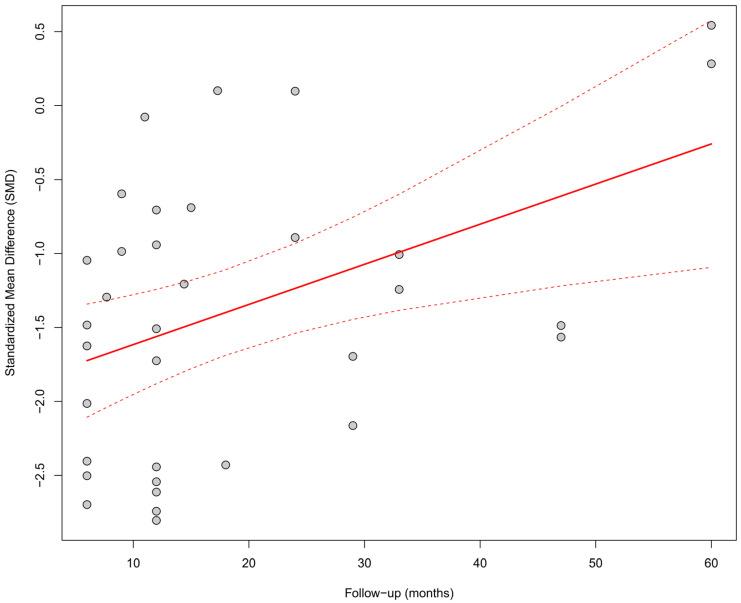
Meta-regression (bubble plot) of the relationship between follow-up duration (in months) and the SMD in LVEF. A significant impact of follow-up time on the observed improvement was found; longer follow-up was associated with a greater increase in LVEF (*β* = +0.027; *p* = 0.0055), and the model explained 18.3% of the total heterogeneity [21,30,31,32,34,35,36,37,38,39,41,43,44,45,48,49,50,51,53,55].

Meta-regression assessed the relationship between the duration of follow-up and the effect size. A significant impact of follow-up time on the observed improvement was found; longer follow-up was associated with a greater increase in LVEF (*β* = +0.027; *p* = 0.0055), and the model explained 18.3% of the total heterogeneity. Figure 4 presents a bubble plot, where the size of the points reflects the precision of the estimate, and the upward trend is visualized as a positively sloped regression line.

The classic funnel plot showed no clear asymmetry, which was confirmed by Egger’s test (*p* = 0.128), indicating a low risk of publication bias. Nevertheless, visual assessment suggested a possible underrepresentation of studies with neutral outcomes. The application of the trim-and-fill method indicated the potential presence of nine missing analyses on the positive side of the distribution, resulting in a shift of the cumulative effect to an SMD of −0.98 (95%CI: −1.33 to −0.63). This effect remained statistically significant, confirming its robustness even after accounting for a possible influence of publication bias.

Based on the data collected from the analyzed studies, physiological pacing was associated with a significant improvement in LVEF compared to baseline. This effect was observed both in the overall analysis and in the subgroup analysis, with the greatest improvement in LVEF seen after LBBAP implantation, slightly smaller after HBP, while in the case of conventional pacing (BVP, RVP), the effect was weaker or not significant.

### 3.2. Effects on Left Ventricular End-Diastolic Volume (LVEDV)

The analysis of the impact of different cardiac pacing techniques on LVEDV was conducted based on nine comparisons derived from five publications. Overall, the implantation of a pacing system was associated with a significant reduction in LVEDV—the pooled SMD was 0.66 (95%CI: 0.25 to 1.08; *p* = 0.0015), as illustrated in Figure S2. However, significant heterogeneity was observed among the studies (I^2^ = 90.0%), indicating substantial variability in the results and justifying further stratified analyses.

**Figure 5:**
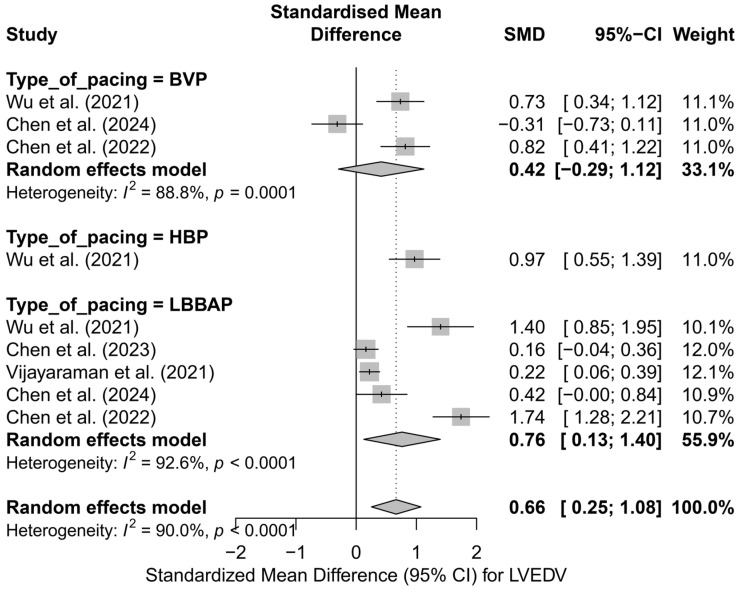
SMD in LVEDV according to the type of pacing—subgroup analysis (BVP, HBP, LBBAP) in the random-effects model. The strongest effect was observed for physiological techniques (LBBAP, HBP) [32,33,34,48,53].

The subgroup analysis according to the applied pacing technique revealed that the most pronounced reduction in LVEDV occurred in LBBAP—SMD = 0.76 (95%CI: 0.13 to 1.40; I^2^ = 92.6%) and HBP—SMD = 0.97 (95%CI: 0.55 to 1.39; based on a single study). For conventional BVP pacing, the effect was lower and did not reach statistical significance (SMD = 0.42; 95%CI: −0.29 to 1.12; I^2^ = 88.8%). The differences between techniques did not reach the threshold for statistical significance (*Q* = 1.77; *p* = 0.412), although a trend favoring physiological techniques was observed. A graphical representation of this analysis is presented in Figure 5.

**Figure 6:**
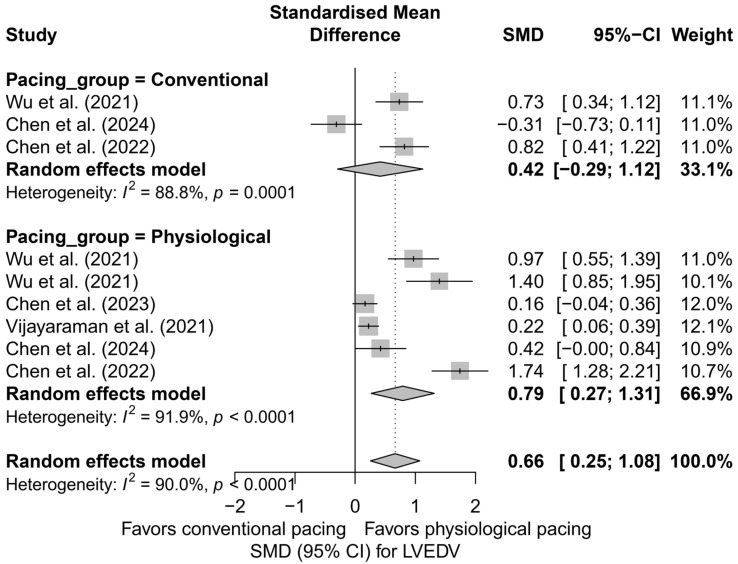
SMD in LVEDV according to the type of pacing—physiological (HBP, LBBAP) vs. conventional (BVP); random-effects model. Physiological pacing was associated with a greater reduction in LVEDV compared to conventional pacing [32,33,34,48,53].

A further comparison combining physiological techniques (LBBAP and HBP) with conventional pacing (BVP) also favored physiological pacing. In the physiological group, a significant reduction in LVEDV was demonstrated (SMD = 0.79; 95%CI: 0.27 to 1.31; I^2^ = 91.9%), whereas for conventional pacing, the result was not statistically significant (SMD = 0.42; 95%CI: −0.29 to 1.12; I^2^ = 88.8%). Despite clear differences in the estimates, the test comparing the groups did not reach statistical significance (*Q* = 0.70; *p* = 0.404), which may be due to the small number of analyses and substantial heterogeneity. A comparative plot of the physiological and conventional groups is presented in Figure 6.

The head-to-head comparison analysis, including only studies that compared more than one type of pacing within the same publication (*k* = 7), demonstrated a significant reduction in LVEDV: SMD = 0.82 (95%CI: 0.33 to 1.31; *p* = 0.0011), with high heterogeneity (I^2^ = 88.2%). When stratified by pacing technique, the highest effect was observed for LBBAP (SMD = 1.18; 95%CI: 0.39 to 1.97), and the lowest for BVP (SMD = 0.42; 95%CI: −0.29 to 1.12). However, it should be emphasized that this was not a classical analysis of differences between techniques in a direct comparison model, but rather an analysis of the aggregated effect for each technique within multi-arm studies. Therefore, the results support the advantage of physiological techniques, although they cannot be considered as definitive evidence of the direct superiority of one technique over another.

In the conducted meta-regression including follow-up time, no significant relationship was found between the duration of follow-up and the effect size (*β* = −0.0445; *p* = 0.233; R^2^ = 3.5%). The trend suggested a slight reduction in the effect with longer follow-up, but it did not reach the threshold of statistical significance.

Finally, the risk of publication bias was assessed. In the classic funnel plot, a visible asymmetry in the distribution was observed, which may suggest the presence of publication bias, particularly a lack of analyses with low effect sizes and small SE on the left side. Due to the limited number of available analyses (*k* = 9), a formal Egger’s test was not performed. The application of the trim-and-fill method indicated the potential presence of three missing analyses, which, after imputation, resulted in a weakening of the cumulative effect to an SMD of 0.30 (95%CI: −0.19 to 0.80; *p* = 0.233). As a result, the effect was no longer statistically significant. This finding suggests that the true impact of pacing on LVEDV may be lower than originally estimated, which should be taken into account in clinical interpretation and in the planning of future studies.

The analysis of LVEDV demonstrated a trend toward its reduction following the use of CSP. The greatest decrease in LVEDV was observed after LBBAP implantation, whereas in the BVP groups, the effect was less pronounced.

### 3.3. Effects on Left Ventricular End-Systolic Volume (LVESV)

In the analysis of LVESV, 13 comparisons derived from seven publications were included. The overall effect in the random-effects model demonstrated a significant reduction in LVESV following cardiac pacing, with an SMD of 1.14 (95%CI: 0.81 to 1.47, *p* < 0.0001). Significant heterogeneity was observed between studies (I^2^ = 87.0%), which was also confirmed by the tau^2^ value and the Q-test (*p* < 0.0001). The results are presented in Figure S3, where a shift of the effects toward a significant improvement in left ventricular systolic function, measured by the reduction in LVESV, is clearly visible.

**Figure 7:**
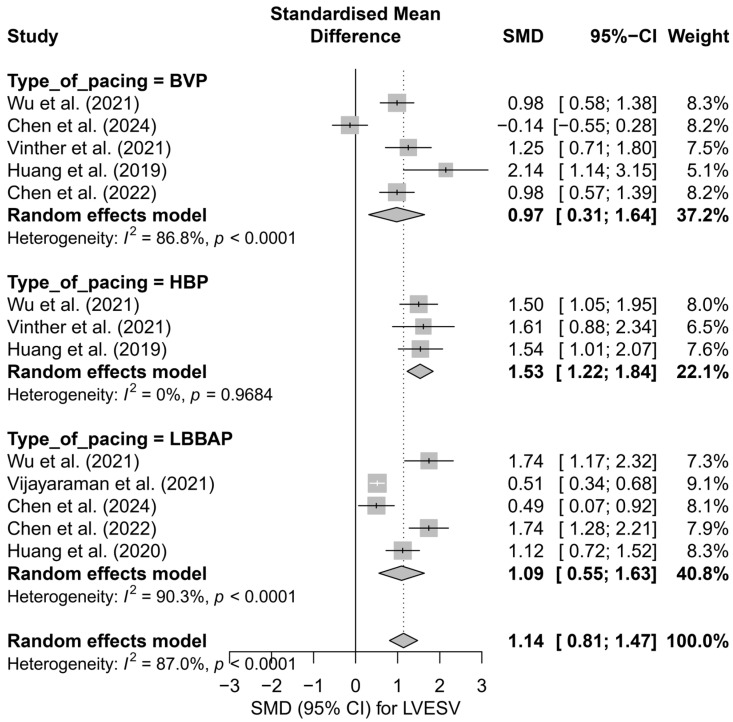
Forest plot of the subgroup analysis of the impact of pacing techniques (BVP, HBP, LBBAP) on LVESV. The differences between techniques were not statistically significant (*p* = 0.157) [32,34,37,38,48,50,53].

The subgroup analysis based on the type of pacing technique is illustrated in Figure 7. The greatest effect was observed for HBP (SMD = 1.53; 95%CI: 1.22 to 1.84), with no heterogeneity (I^2^ = 0%). LBBAP also showed a significant, moderate effect (SMD = 1.09; 95%CI: 0.55 to 1.63), although with very high variability between studies (I^2^ = 90.3%). The smallest effect was seen in the BVP group (SMD = 0.97; 95%CI: 0.31 to 1.64; I^2^ = 86.8%). The differences between techniques did not reach statistical significance (*p* = 0.179), but a noticeable gradient in effect size favored physiological pacing.

**Figure 8:**
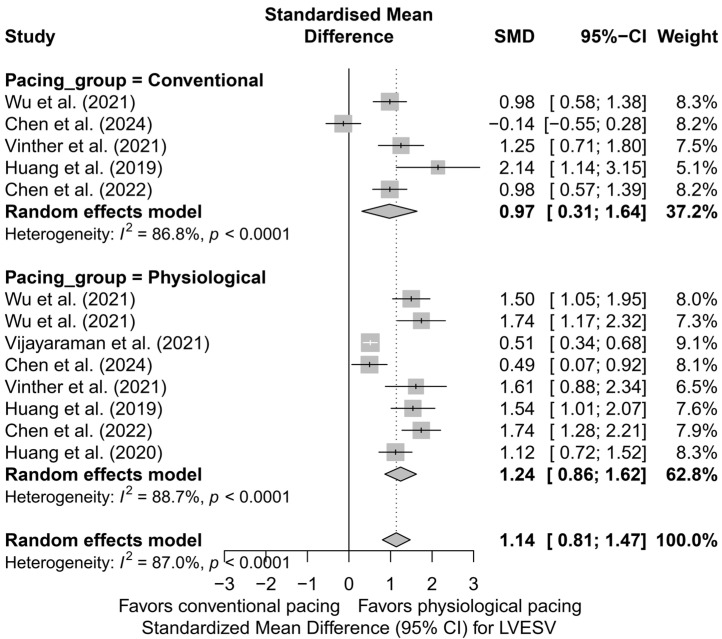
Forest plot comparing the effect of physiological (HBP, LBBAP) and conventional (BVP) cardiac pacing on the reduction in LVESV. The effect was greater in the physiological group (SMD = 1.24; I^2^ = 88.7%) than in the conventional group (SMD = 0.97; I^2^ = 86.8%), although the difference between groups was not statistically significant (*p* = 0.492) [32,34,37,38,48,50,53].

Similar observations were obtained in the analysis directly comparing physiological and conventional pacing, presented in Figure 8. The effect of LVESV reduction was more pronounced for physiological techniques (SMD = 1.24; 95%CI: 0.86 to 1.62; I^2^ = 88.7%) than for conventional BVP (SMD = 0.97; 95%CI: 0.31 to 1.64; I^2^ = 86.8%). This difference did not reach statistical significance (*p* = 0.492), but the direction and magnitude of the clinical effect remain consistent with the analysis based on pacing technique.

In a single study enabling a head-to-head comparison between HBP and LBBAP [53], both techniques demonstrated substantial efficacy in reducing LVESV (HBP: SMD = 1.50; LBBAP: SMD = 1.74). The mean effect for this comparison was SMD = 1.59 (95%CI: 1.24 to 1.95), with no detected heterogeneity (I^2^ = 0%). The difference between the techniques was not statistically significant (*p* = 0.514), which may be due to the limitation of having only one data source.

The meta-regression analysis assessed the impact of follow-up duration on the effect size of therapy. A trend was observed indicating a gradual decrease in effect size with longer follow-up (*β* = −0.0522; *p* = 0.0511), with heterogeneity explained at the level of R^2^ = 19.8%. This result is at the threshold of statistical significance and may reflect processes of late remodeling or the influence of confounding factors present in longer follow-up periods.

The analysis of publication asymmetry using Egger’s test revealed significant funnel plot asymmetry (*p* = 0.0099), suggesting the presence of publication bias favoring studies with positive results. The application of the trim-and-fill method, which added hypothetical missing studies to the left side of the distribution, led to a reduction of the estimated effect to an SMD of 0.67 (95%CI: 0.27 to 1.08; *p* = 0.0011). Despite this correction, the effect remained statistically significant, which strengthens its credibility, although it should be taken into account in clinical interpretation.

The use of CSP was also associated with a reduction in LVEDV. In the subgroup analyses, LBBAP demonstrated the most significant improvement in this parameter, indicating a favorable effect on left ventricular remodeling.

### 3.4. Effects on QRS Duration

In the meta-analysis including 41 comparisons from 24 publications, the impact of different cardiac pacing techniques on QRS duration was assessed. In the overall analysis, a significant effect of QRS shortening was found in the intervention groups compared to the control groups, with a pooled effect of SMD = 0.75 (95%CI: 0.24 to 1.26; *p* = 0.0042). Heterogeneity was very high (I^2^ = 98.5%), indicating substantial variability among the studies (Figure S4).

**Figure 9:**
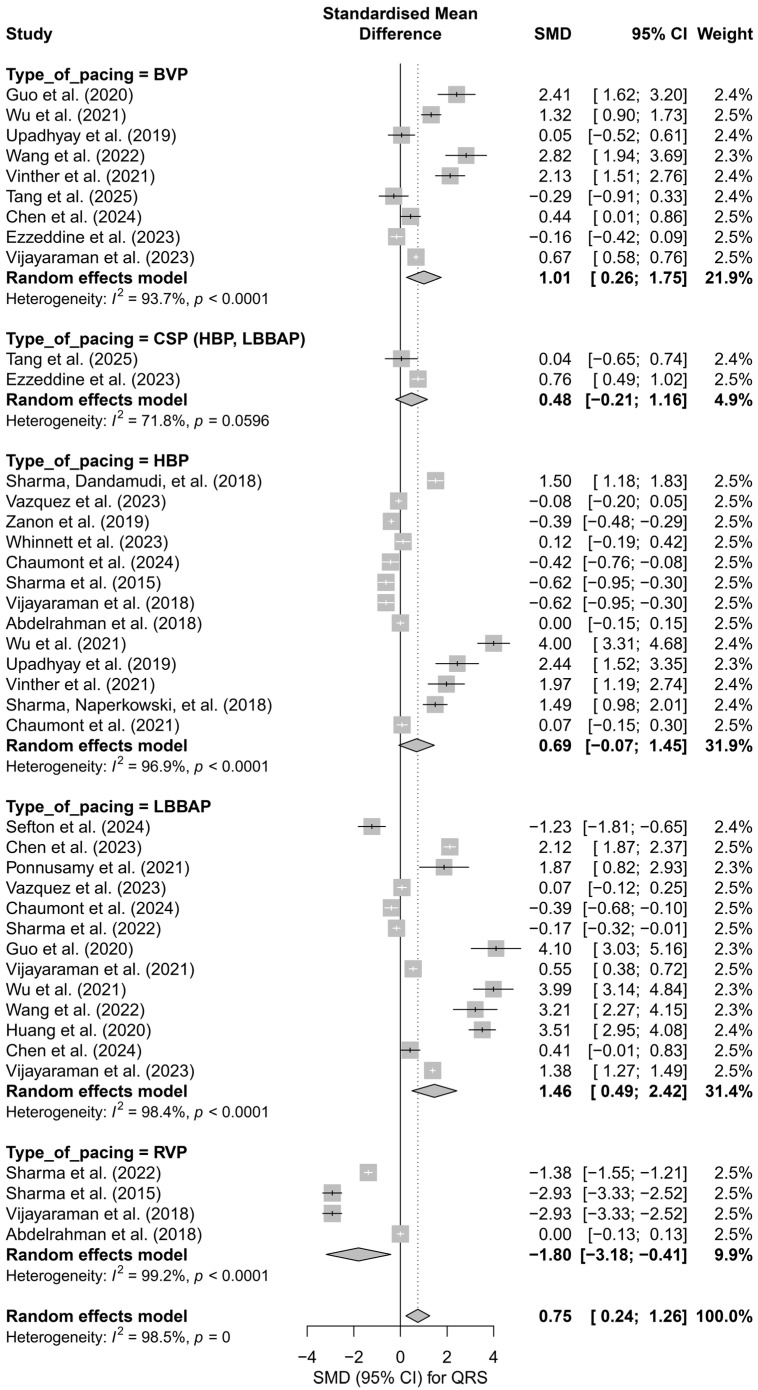
Forest plot presenting the effect of different cardiac pacing techniques on QRS duration. The greatest QRS shortening was observed for LBBAP (SMD = 1.46) and HBP (SMD = 0.69), whereas RVP led to QRS prolongation (SMD = −1.80). The effects for BVP (SMD = 1.01) and CSP (SMD = 0.48) were smaller, with the CSP group not reaching statistical significance. Test for differences between subgroups: *Q* = 15.87, *df* = 4, *p* = 0.0032. Note: the *p*-value for heterogeneity (*p* = 0) was rounded by the RStudio environment; the actual *p*-value is less than 0.0001 [15,21,30,31,33,34,35,36,38,39,40,41,42,43,44,45,46,47,48,49,50,51,52,53].

Subgroup analysis according to the type of pacing showed that the greatest QRS shortening effect was observed with LBBAP (SMD = 1.46; 95%CI: 0.49 to 2.42) and HBP (SMD = 0.69; 95%CI: −0.07 to 1.45), while RVP pacing was associated with a significant prolongation of the QRS complex (SMD = −1.80; 95%CI: −3.18 to −0.41). The I^2^ values for the individual groups were high (highest for RVP—99.2%), and the test for differences between subgroups showed statistical significance (*Q* = 15.87, *df* = 4, *p* = 0.0032), suggesting that the type of pacing significantly influences changes in QRS duration (Figure 9).

**Figure 10:**
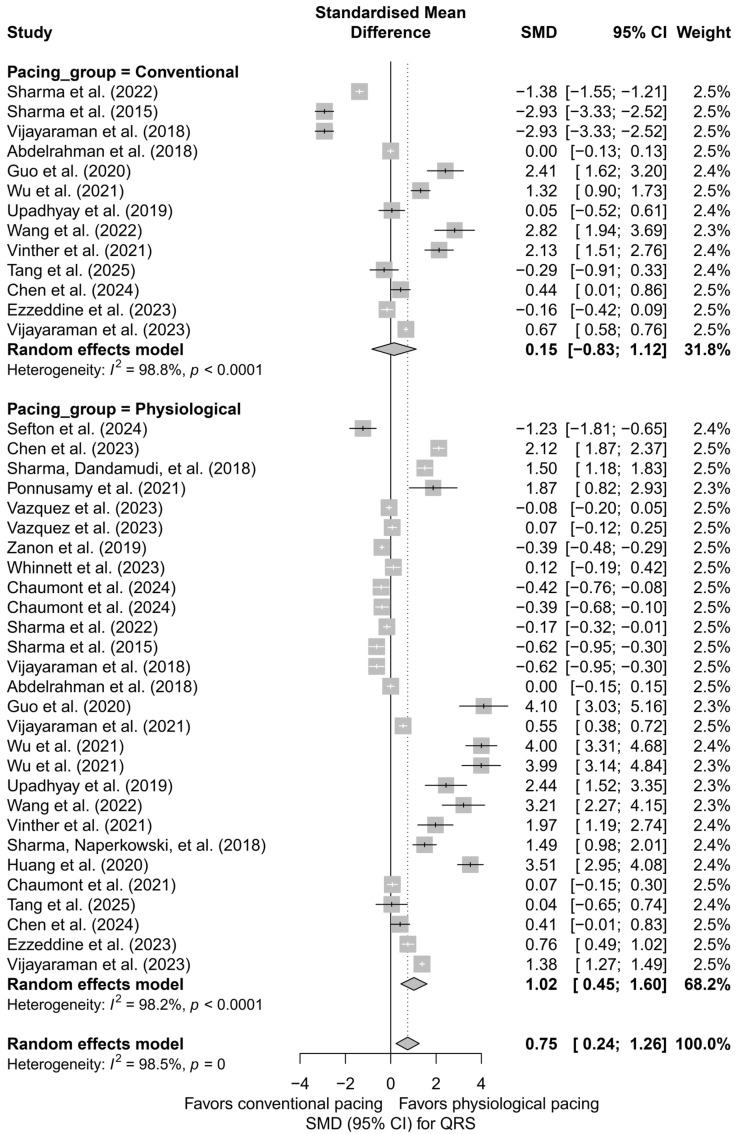
Comparison of physiological and conventional cardiac pacing in the context of their impact on QRS duration. The forest plot presents the meta-analysis of SMD effects for physiological techniques (HBP, LBBAP, CSP) and conventional techniques (BVP, RVP), analyzed separately using a random-effects model. The difference between groups did not reach statistical significance (*Q* = 2.29; *p* = 0.1301). Note: the *p*-value for heterogeneity (*p* = 0) was rounded by the RStudio environment; the actual *p*-value is less than 0.0001 [15,21,30,31,33,34,35,36,38,39,40,41,42,43,44,45,46,47,48,49,50,51,52,53].

In a separate analysis comparing physiological techniques (HBP, LBBAP, CSP) with conventional techniques (BVP, RVP), a moderate and significant QRS shortening effect was found in the physiological group (SMD = 1.02; 95%CI: 0.45 to 1.60; I^2^ = 98.2%). In the conventional group, the effect was not statistically significant (SMD = 0.15; 95%CI: −0.83 to 1.12; I^2^ = 98.8%). The difference between groups did not reach the threshold for statistical significance (*Q* = 2.29, *df* = 1, *p* = 0.1301), but the trend suggests an advantage of physiological pacing in terms of electrophysiological synchronization (Figure 10).

For studies with direct comparisons of techniques (head-to-head analysis, *k* = 31), a similar effect to the overall analysis was obtained: SMD = 0.68 (95%CI: 0.06 to 1.31; *p* = 0.0312), again with very high heterogeneity (I^2^ = 98.5%). In the subgroups, the effect was highest for LBBAP (SMD = 1.52) and HBP (SMD = 0.80), while RVP continued to show a negative impact on QRS duration (SMD = −1.80). The test for differences between subgroups confirmed statistical significance (*Q* = 14.85, *df* = 4, *p* = 0.0050).

**Figure 11:**
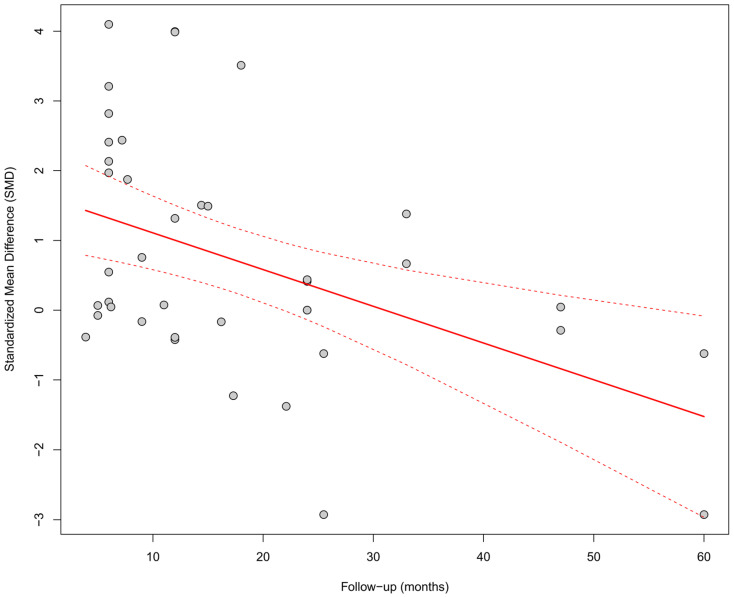
Meta-regression assessing the relationship between follow-up duration and the effect size of cardiac pacing on QRS duration. Each point represents an individual study, and the regression line (with 95%CI) shows a significant downward trend (*p* = 0.0013). As the follow-up period increased, the effect of QRS shortening (SMD) weakened, which may suggest a decreasing effectiveness over time [15,21,30,31,33,34,35,36,38,39,40,41,42,43,44,45,46,47,48,49,50,51,52,53].

In the meta-regression including follow-up duration (*k* = 40), a significant inverse relationship was observed between follow-up length and the SMD effect size (*β* = −0.0527, SE = 0.0164, *p* = 0.0013, R^2^ = 19.5%). This result suggests that the beneficial effect of physiological pacing on QRS shortening may diminish over time (Figure 11).

In the publication bias analysis, no significant funnel plot asymmetry was detected according to Egger’s test (*p* = 0.2748); however, visual inspection revealed a lack of smaller studies with negative effects. Further analysis using the trim-and-fill method suggested the presence of seven potentially missing studies, and the adjusted effect was no longer statistically significant (SMD = 0.21; 95%CI: −0.37 to 0.79, *p* = 0.475), indicating the possibility of an overestimation of the original effect.

Physiological pacing led to a significant shortening of QRS duration. The greatest reduction was observed in the LBBAP group, while HBP and CSP were also associated with a marked narrowing of the QRS complex. Conventional techniques did not demonstrate a comparable effect.

### 3.5. Effects on NYHA Functional Class

The analysis including 19 comparisons from 11 publications demonstrated a clear improvement in the NYHA functional class following the implantation of a pacing system, regardless of the technique used. For ease of interpretation, clinical improvement (decrease in NYHA class) was presented as a positive SMD value. In the random-effects model, a significant overall effect was obtained (SMD = 1.94; 95%CI: 1.59 to 2.29; *p* < 0.0001), with very high heterogeneity among studies (I^2^ = 96.5%). These results are illustrated in Figure S5, presenting the forest plot for all analyzed comparisons.

**Figure 12:**
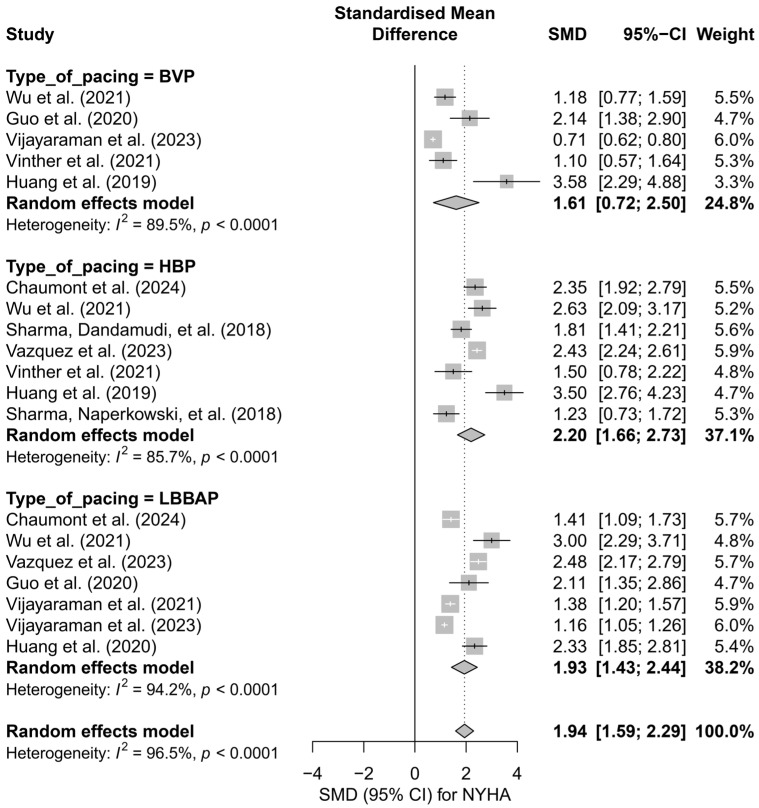
Meta-analysis of the effect of cardiac pacing on NYHA class—subgroup analysis according to pacing type. The forest plot presents the clinical effects (SMD, 95%CI) for three pacing techniques: BVP, HBP, and LBBAP. Positive SMD values indicate improvement in NYHA class. Differences between subgroups were not statistically significant (*p* = 0.52) [31,36,37,38,43,44,47,48,49,50,53].

In the subgroup analysis according to pacing technique (BVP, HBP, LBBAP), a significant improvement in the NYHA class was observed across all three groups: the greatest effect was recorded for HBP (SMD = 2.20; 95%CI: 1.66 to 2.73), and the smallest for BVP (SMD = 1.61; 95%CI: 0.72 to 2.50). However, the differences between techniques did not reach statistical significance (*Q* = 1.31; *p* = 0.519), as shown in Figure 12.

**Figure 13:**
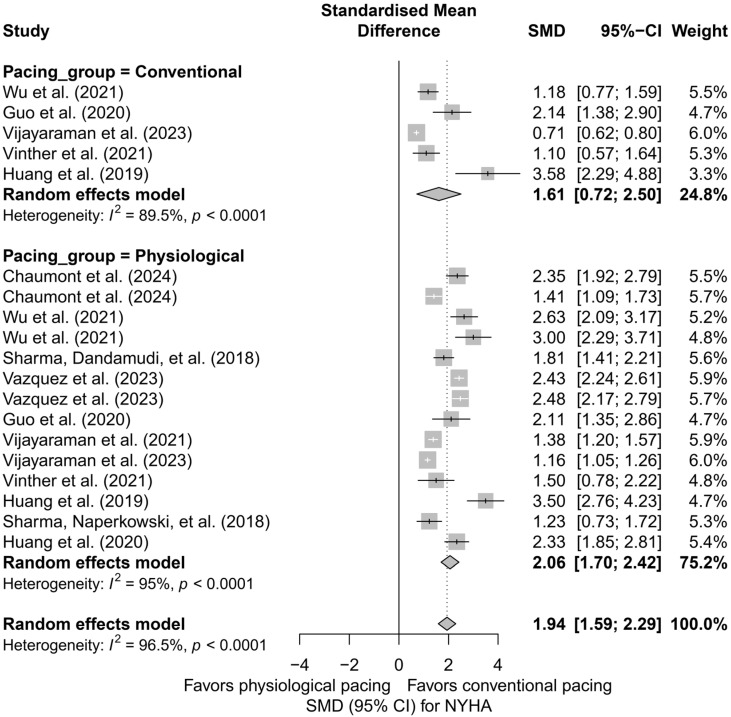
Meta-analysis of the effect of physiological versus conventional pacing on NYHA class. The forest plot presents the clinical effect (SMD, 95%CI) in two groups: conventional pacing (BVP, RVP) and physiological pacing (HBP, LBBAP). The improvement in NYHA class was greater in the physiological group (SMD = 2.06 vs. 1.61), although the difference between groups did not reach statistical significance (*p* = 0.36) [31,36,37,38,43,44,47,48,49,50,53].

Similar conclusions arise from the analysis comparing physiological pacing (HBP, LBBAP) with conventional pacing (BVP, RVP). The effect was greater in the physiological group (SMD = 2.06; 95%CI: 1.70 to 2.42) than in the conventional group (SMD = 1.61; 95%CI: 0.72 to 2.50), although this difference was not statistically significant (*Q* = 0.84; *p* = 0.36). The results are summarized in Figure 13.

In the head-to-head analysis, limited to studies including ≥two pacing techniques, no significant differences were found between HBP and LBBAP (*Q* = 0.14; *p* = 0.7074), although HBP was associated with a slightly greater and more homogeneous improvement in the NYHA class (SMD = 2.44; I^2^ = 0%) compared to LBBAP (SMD = 2.26; I^2^ = 93.3%).

The meta-regression analysis with follow-up time as a moderating variable showed a trend suggesting that longer follow-up may be associated with a smaller clinical effect (*β* = −0.0356; SE = 0.0199; *p* = 0.073), with 15.3% of the heterogeneity explained by this variable.

The publication bias analysis revealed significant asymmetry in the funnel plot (Egger’s test: *p* = 0.0027), which may indicate the presence of publication bias. After applying the trim-and-fill correction, the estimated effect was reduced to an SMD of 1.09 (95%CI: 0.57 to 1.61), although it remained statistically significant (*p* < 0.0001).

The analysis of NYHA functional class demonstrated a substantial improvement in the clinical status of patients after the implantation of CSP systems. The most pronounced therapeutic effect was observed in the HBP and LBBAP groups. In the analyzed studies, no cases of NYHA class deterioration following physiological pacing were reported.

### 3.6. Effects on Brain Natriuretic Peptide (BNP) Levels

This meta-analysis included five study arms derived from three independent publications. The overall analysis demonstrated a significant and clinically meaningful reduction in BNP levels following the implantation of a pacing system—the SMD was 0.91 (95%CI: 0.63 to 1.19; *p* < 0.0001), indicating a moderate to large effect. Heterogeneity was zero (*I*^2^ = 0%), suggesting exceptional consistency of results across studies, despite differences in pacing techniques and potential differences in BNP measurement units. The effects are presented in Figure S6.

**Figure 14:**
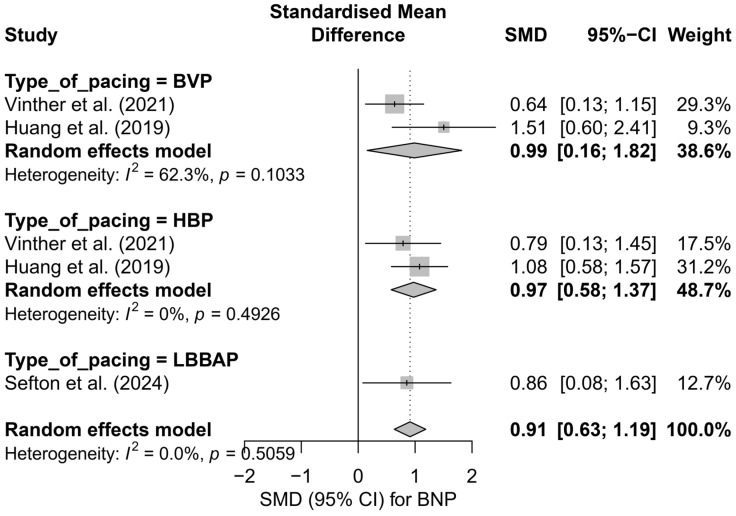
Forest plot presenting the results of the meta-analysis stratified by pacing type: BVP, HBP, and LBBAP. The overall effect for the entire group was SMD = 0.91 [0.63; 1.19]. The absence of significant differences between pacing types (*Q* = 0.08, *p* = 0.776) suggests a comparable impact of these techniques on BNP levels [37,41,50].

In the subgroup analysis, presented in Figure 14, all types of pacing demonstrated a directionally similar effect. In the HBP subgroup, high consistency and a moderate effect were observed (SMD = 0.97; 95%CI: 0.58 to 1.37; I^2^ = 0%). BVP was characterized by slightly greater variability in results (SMD = 0.99; 95%CI: 0.16 to 1.82; I^2^ = 62.3%), whereas for LBBAP, only one study arm was available (SMD = 0.86; 95%CI: 0.08 to 1.63), making it impossible to estimate heterogeneity. The test for differences between subgroups did not show statistical significance (*Q* = 0.08; *p* = 0.776), indicating the comparable effectiveness of different techniques in reducing BNP levels.

**Figure 15:**
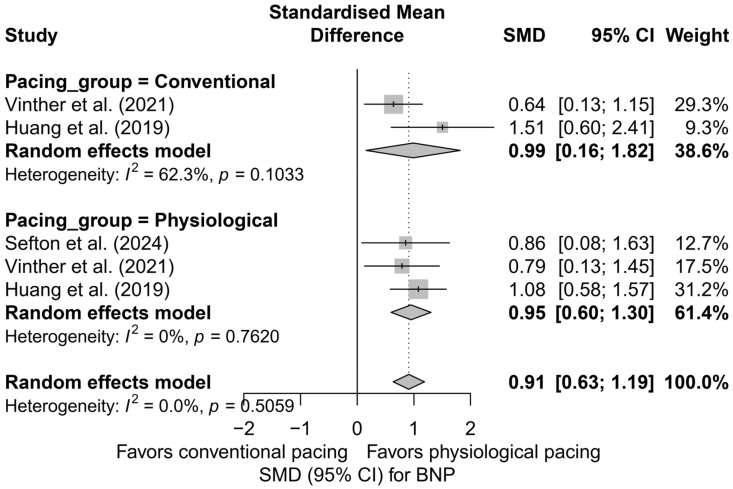
Forest plot comparing the impact of physiological pacing (HBP and LBBAP) with conventional (BVP) on BNP levels. Both strategies were associated with a significant reduction in BNP. The test for differences between groups was not statistically significant (*Q* = 0.01, *p* = 0.933), suggesting comparable effectiveness of both approaches in this regard [37,41,50].

In the comparison between physiological pacing (HBP and LBBAP) and conventional pacing (BVP), no significant differences were found (Figure 15). Both groups showed a significant reduction in BNP levels—physiological pacing: SMD = 0.95 (95%CI: 0.60 to 1.30; I^2^ = 0%), conventional pacing: SMD = 0.99 (95%CI: 0.16 to 1.82; I^2^ = 62.3%). The absence of a statistically significant difference between groups (*Q* = 0.01; *p* = 0.933) suggests the equivalence of the effect in terms of improvement in hemodynamic parameters assessed by BNP levels.

The head-to-head analysis was limited to four study arms from two publications containing multi-arm comparisons. In this case as well, no significant differences were found between HBP and BVP (SMD = 0.97 vs. 0.99; *p* = 0.976), further confirming the equivalence of the BNP reduction effect between these strategies.

Meta-regression with respect to follow-up duration showed a trend toward an increasing effect over time; however, this relationship did not reach statistical significance (*p* = 0.3149). The range of effect sizes was wide, which should be interpreted with caution due to the small number of comparisons (*k* = 5) and the limited statistical power.

The publication bias analysis did not reveal any asymmetry suggesting the presence of publication bias. The distribution of points in the funnel plot was symmetrical relative to the vertical axis, and the trim-and-fill method did not suggest the need for imputing missing studies (number of added studies: 0; corrected effect: SMD = 0.91; 95%CI: 0.63 to 1.19). Egger’s test was not performed due to the small number of analyzed study arms (*k* = 5).

Available data suggest a significant reduction in BNP levels following the implantation of CSP systems, which may reflect a beneficial impact of physiological pacing on left ventricular filling pressures. The effect was particularly evident in the LBBAP and HBP groups.

### 3.7. Effects on R-Wave Amplitude

In the meta-analysis including 14 comparisons from nine publications, a small but statistically significant reduction in R-wave amplitude was observed after the implantation of cardiac pacing systems. The mean adjusted effect, SMD, was −0.16 (95%CI: −0.26 to −0.06; *p* = 0.0024), corresponding to a mild decrease in R-wave signal amplitude during post-implantation follow-up. Heterogeneity between study results was assessed as moderate (I^2^ = 43.8%). A detailed distribution of effects is presented in Figure S7.

**Figure 16:**
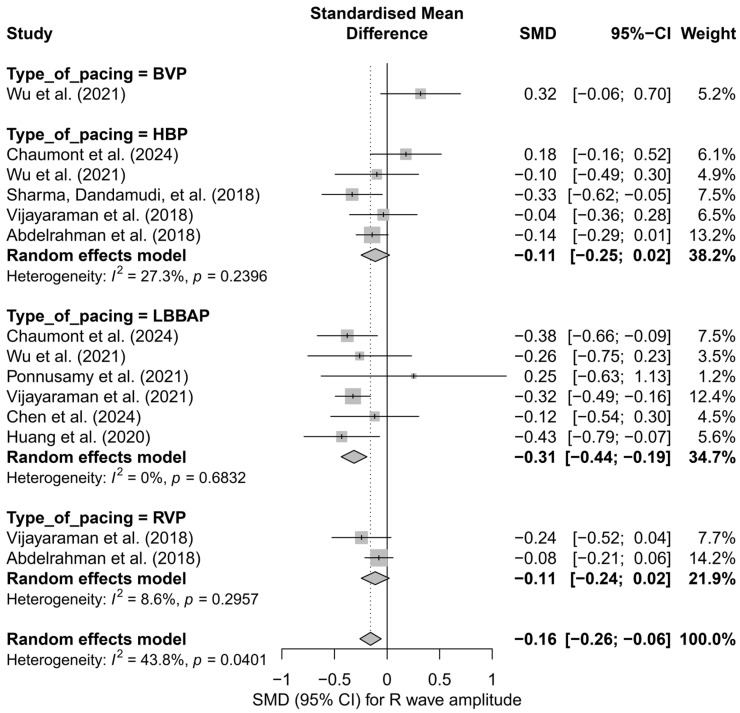
Forest plot showing the change in R-wave amplitude after implantation of pacing systems, stratified by pacing type. For each technique (BVP, HBP, LBBAP, RVP), the pooled meta-analytic effect was presented using a random-effects model. A significant reduction in amplitude was observed only in the LBBAP group. In the remaining subgroups, the effects were small and not statistically significant. Differences between groups were statistically significant (subgroup test: *p* = 0.0044) [15,21,31,34,38,39,43,48,53].

In the subgroup analysis considering the type of pacing (HBP, LBBAP, RVP, BVP), it was shown that the most pronounced and statistically significant decrease in R-wave amplitude occurred in the LBBAP group (SMD = −0.31; 95%CI: −0.44 to −0.19; *p* < 0.01; I^2^ = 0%). In the cases of HBP and RVP, the decreases were mild and did not reach statistical significance (HBP: SMD = −0.11; RVP: SMD = −0.11). Interestingly, the only study in the BVP group showed an opposite trend—a non-significant increase in amplitude (SMD = +0.32). The differences between subgroups were statistically significant (*Q* = 13.13; *p* = 0.0044), indicating a genuine influence of the pacing type on changes in R-wave signal amplitude (Figure 16).

**Figure 17:**
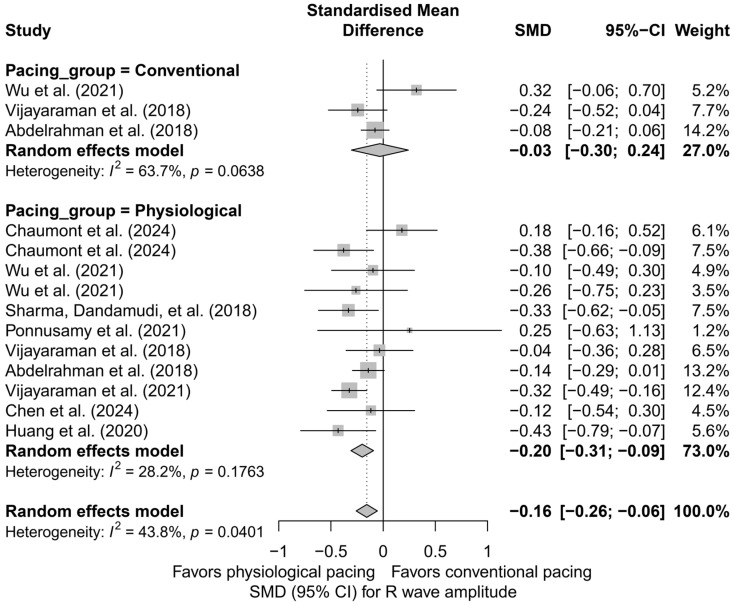
Change in R-wave amplitude stratified by physiological (HBP, LBBAP) and conventional (RVP, BVP) pacing groups. The analysis revealed a significant decrease in amplitude only in the physiological pacing group. The effect in the conventional group was minimal and not statistically significant. Although the test for differences between groups did not reach statistical significance (*p* = 0.259), the plot suggests that the reduction in R-wave amplitude may be predominantly associated with physiological pacing techniques [15,21,31,34,38,39,43,48,53].

A further comparison of physiological techniques (HBP, LBBAP) versus conventional techniques (RVP, BVP) revealed that a significant reduction in R-wave amplitude occurred only in the physiological group (SMD = −0.20; 95%CI: −0.31 to −0.09; I^2^ = 28.2%), while the effect in the conventional group was minimal and not statistically significant (SMD = −0.03; 95%CI: −0.30 to 0.24; I^2^ = 63.7%). Although the difference between these two groups did not reach the threshold for statistical significance (*Q* = 1.27; *p* = 0.259), the direction of change suggests that the decrease in R-wave amplitude is primarily characteristic of physiological pacing techniques (Figure 17).

In the head-to-head analysis, which included nine study arms from publications comparing more than one pacing technique, the adjusted overall effect was SMD = −0.10 (95%CI: −0.20 to +0.01; *p* = 0.063), which was borderline non-significant. In the LBBAP subgroup, the effect was clear and statistically significant (SMD = −0.35; 95%CI: −0.60 to −0.10), whereas in the remaining groups—HBP, RVP, and BVP—the effects were not statistically significant (HBP: SMD = −0.07; RVP: SMD = −0.11; BVP: SMD = +0.32). The differences between pacing techniques in this direct comparison also reached statistical significance (*Q* = 8.80; *p* = 0.0321), further confirming the distinct impact of individual strategies on recorded R-wave amplitude.

Meta-regression analysis with respect to follow-up duration showed no association between the length of follow-up and the magnitude of change in R-wave amplitude (*β* = 0.0007; *p* = 0.825). The regression line was nearly flat, and the wide CIs crossed the zero axis, indicating that any observed changes are likely to occur early and do not progress over time.

In assessing the risk of publication bias, the funnel plot showed no significant asymmetry, and Egger’s test confirmed the absence of statistically significant bias (*p* = 0.648). After applying the trim-and-fill method, one potentially missing study was imputed on the left side of the plot, which did not materially affect the overall result—the effect remained statistically significant (SMD = −0.18; 95%CI: −0.29 to −0.07; *p* = 0.0016), confirming the robustness of the finding even when accounting for potential publication asymmetry.

Additionally, from a clinical practice perspective, the observed decrease in R-wave amplitude, particularly in the LBBAP group, may have implications for pacing safety and sensing margin. Although absolute mean amplitude differences between techniques were not analyzed, the negative SMD for LBBAP relative to HBP may suggest a more pronounced signal reduction over time. These findings should be considered when selecting the implantation technique and evaluating the long-term performance of the pacing lead.

### 3.8. Effects on Pacing Thresholds

The analysis of the impact of different pacing techniques on pacing threshold included 17 comparisons from 11 publications. This parameter holds significant clinical relevance, as it reflects both long-term battery consumption and the durability and efficiency of lead performance. The initial overall analysis showed that differences between pacing techniques were not statistically significant (SMD = −0.14; 95%CI: −0.36 to 0.08; *p* = 0.22), and the high heterogeneity (I^2^ = 81.9%) indicated considerable variability between studies (Figure S8). Therefore, further subgroup analyses and meta-regression were conducted.

**Figure 18:**
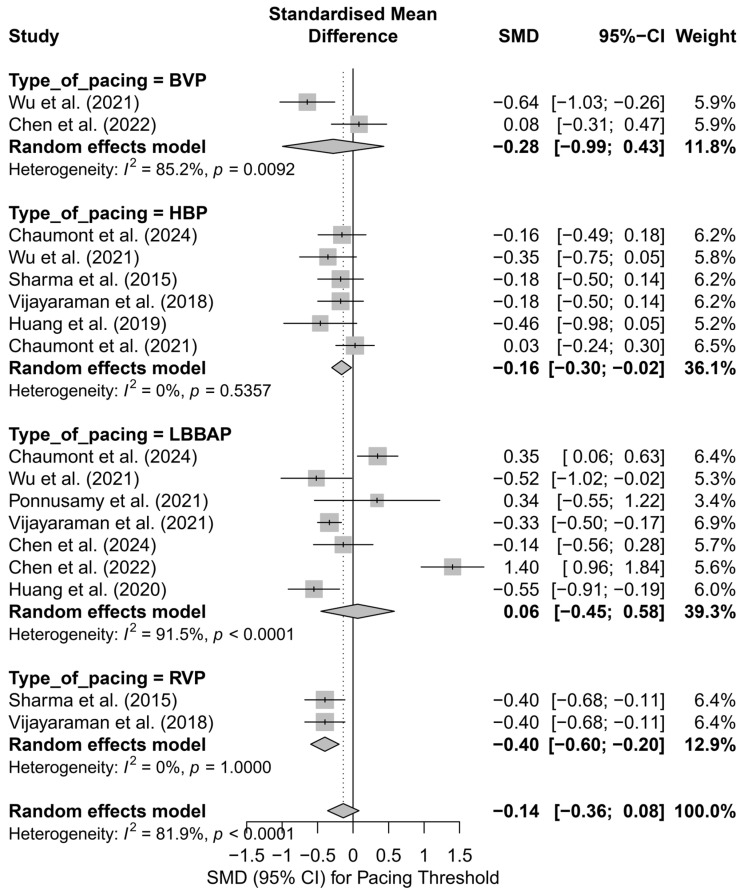
Forest plot presenting the SMD in pacing threshold values stratified by technique: HBP, LBBAP, BVP, and RVP. Significantly lower pacing thresholds were observed in the HBP and RVP groups (SMD = −0.16 and SMD = −0.40, respectively), both with no heterogeneity (I^2^ = 0%). In the BVP and LBBAP groups, the effects were not statistically significant, with high variability observed in the LBBAP group (I^2^ = 91.5%). The test for differences between techniques did not reach statistical significance (*Q* = 4.88; *p* = 0.18) [21,30,31,32,34,37,38,39,42,48,53].

In the subgroup analysis by pacing technique (Figure 18), only HBP showed a significantly lower pacing threshold compared to control groups (SMD = −0.16; 95%CI: −0.30 to −0.02; I^2^ = 0%). RVP was also associated with a significantly lower threshold (SMD = −0.40; 95%CI: −0.60 to −0.20), though these results should be interpreted with caution due to the limited number of studies. No significant differences were observed for LBBAP or BVP, with very high heterogeneity in the LBBAP group (I^2^ = 91.5%). The test for subgroup differences did not reach statistical significance (*Q* = 4.88; *p* = 0.18), but the evident variability suggests a differential impact of individual pacing techniques.

**Figure 19:**
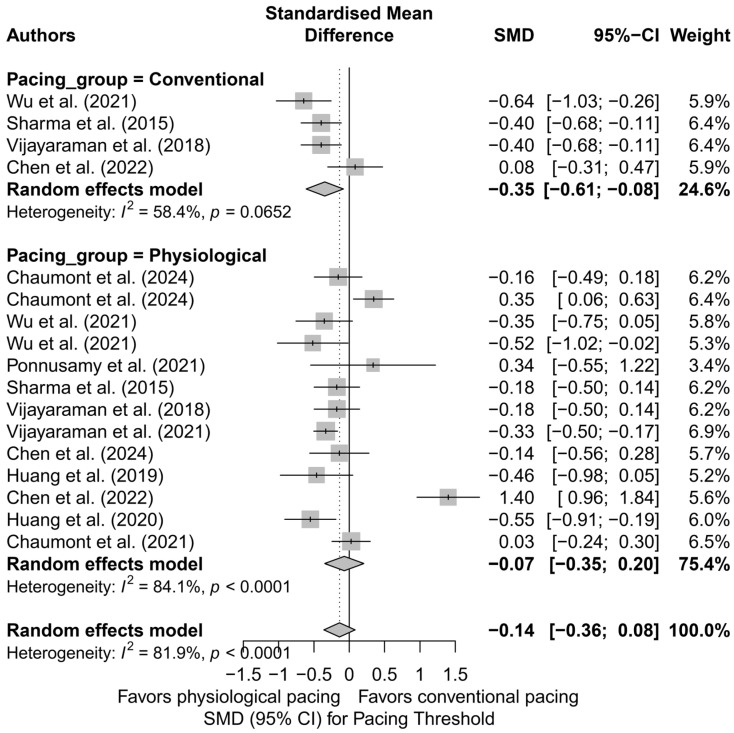
Forest plot presenting the standardized mean difference (SMD) in pacing threshold values stratified by group: physiological pacing (HBP, LBBAP, CSP) and conventional pacing (RVP, BVP). A significantly lower pacing threshold was observed in the conventional group (SMD = −0.35; 95%CI: −0.61 to −0.08; I^2^ = 58.4%), while the effect for physiological pacing did not reach statistical significance (SMD = −0.07; 95%CI: −0.35 to 0.20; I^2^ = 84.1%). Although the difference between groups was not statistically significant (*Q* = 3.17; *p* = 0.075), the observed trend may have clinical relevance [21,30,31,32,34,37,38,39,42,48,53].

When comparing physiological pacing (HBP, LBBAP, CSP) with conventional pacing (BVP, RVP), a trend was observed indicating a significantly lower pacing threshold in the conventional group (SMD = −0.35; 95%CI: −0.61 to −0.08; I^2^ = 58.4%), in contrast to the physiological group, where the effect was not statistically significant (SMD = −0.07; 95%CI: −0.35 to 0.20; I^2^ = 84.1%)—Figure 19. Although the difference between groups did not reach statistical significance (*Q* = 3.17; *p* = 0.075), the observed trend may have clinical relevance, particularly when selecting a lead type for patients requiring long-term pacing therapy.

In the head-to-head analyses, which included 11 comparisons from studies evaluating at least two pacing techniques, the overall effect did not reveal significant differences (SMD = −0.09; 95%CI: −0.42 to 0.23; *p* = 0.57; I^2^ = 86.7%). However, attention should be drawn to the results of direct comparisons between HBP and LBBAP, where HBP showed a trend toward a lower pacing threshold (SMD = −0.24; 95%CI: −0.50 to 0.02), whereas LBBAP was characterized by high variability and a non-significant effect (SMD = −0.06; 95%CI: −0.90 to 0.78; I^2^ = 88.5%). The overall difference between these two techniques was not statistically significant (SMD = −0.14; 95%CI: −0.52 to 0.24; *p* = 0.47), with total heterogeneity estimated at I^2^ = 77.6%.

Meta-regression examined the relationship between follow-up duration and effect size. No significant association was found (*p* = 0.48), and the R^2^ value was 0%, suggesting that follow-up length did not significantly influence the difference in pacing threshold. Residual heterogeneity remained high (I^2^ = 86.4%), confirming the complex nature of the parameter under investigation.

The assessment of publication bias revealed no significant asymmetry—Egger’s test was non-significant (*t* = 0.80; *p* = 0.43), and the trim-and-fill method did not indicate the need for imputing missing studies (0 points added). These findings suggest that publication bias did not have a substantial impact on the estimated effect.

In summary, the analysis of pacing threshold reveals notable differences between techniques. Although the overall effect did not reach statistical significance, HBP and RVP demonstrated an advantage in terms of lower pacing thresholds. In contrast, LBBAP, despite its theoretical benefits, did not show superiority and exhibited considerably greater variability in outcomes. These differences may carry clinical relevance in the context of system longevity and battery consumption, which could influence the choice of pacing technique, particularly in patients requiring long-term resynchronization therapy.

### 3.9. Secondary and Summary Analyses

To complement and systematize the findings presented in Section 3.1, Section 3.2, Section 3.3, Section 3.4, Section 3.5, Section 3.6, Section 3.7 and Section 3.8, an additional set of cross-sectional analyses was performed. The primary rationale for this approach lies in the structure of the available dataset, in which some publications provided multiple entries from the same study population (e.g., separate comparisons of HBP vs. RVP and HBP vs. BVP within the same study). Such data cannot be combined in traditional meta-analyses without the risk of overrepresentation. To address this issue, separate analyses were conducted, including the following:

- Head-to-head comparisons limited to multi-arm studies,
- Synthetic comparisons of CSP versus conventional pacing for each clinical parameter,
- Meta-regressions assessing the impact of follow-up duration,
- Assessment of publication bias using Egger’s test and the trim-and-fill method.

#### 3.9.1. Consolidated Head-to-Head Comparisons Across Parameters

Including only studies with at least two comparative arms (multi-arm studies), meta-analyses of the main effect were conducted for each clinical parameter using a random-effects model. The results of the head-to-head analyses are presented in Table 5.

#### 3.9.2. Summary: Physiological vs. Classical Pacing Across Outcomes

Using the classification of pacing types into physiological (CSP: HBP, LBBAP) and conventional (BVP, RVP) groups, moderated meta-analyses (mixed-effects models) were conducted to compare the effects of CSP versus conventional pacing across all outcome variables. Moderated meta-analyses with “pacing type” (CSP vs. conventional) as a moderator were applied. Data from the entire database were included, with duplicated entries from the same studies removed to avoid overrepresentation. The results are summarized in Table 6.

#### 3.9.3. Meta-Regressions Summary Table

Separate linear meta-regressions were performed for each parameter with respect to follow-up duration. Only for LVEF was a significant positive association observed between follow-up time and effect size (SMD), which may suggest a growing benefit of CSP over time. Parameters showing a follow-up-dependent trend: LVEF—significant trend (*p* < 0.01). The remaining parameters (QRS, NYHA, BNP, LVEDV, LVESV, R-wave amplitude, pacing threshold) did not show a significant association with follow-up duration.

To assess whether follow-up duration (expressed in months) influences the effect size (SMD), separate meta-regressions were conducted for each of the analyzed parameters. The results are summarized in Table 7.

#### 3.9.4. Influence of Follow-Up Duration Across Parameters

**Figure 20:**
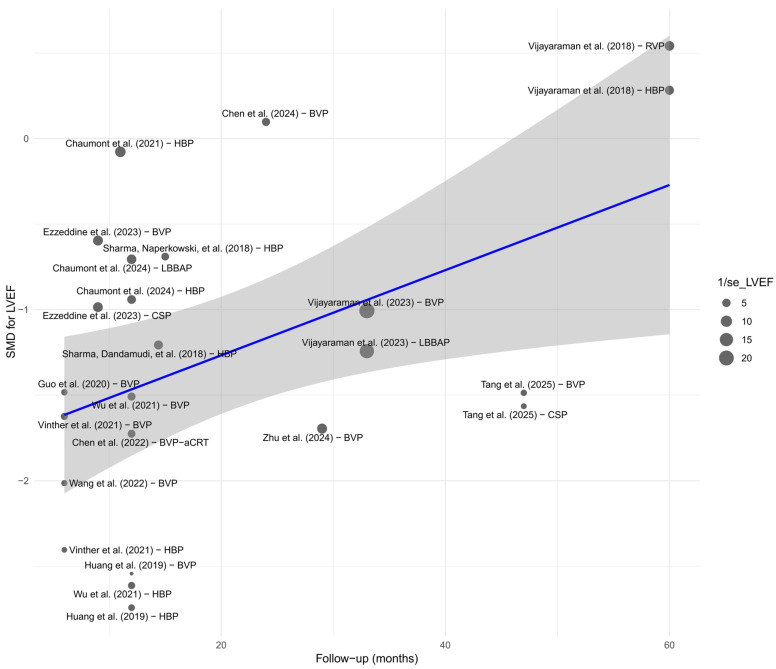
Detailed bubble plot illustrating the relationship between follow-up duration and SMD effect for LVEF. The size of each point corresponds to the precision of the estimate (1/SE). The regression line (in blue), with the 95% Confidence Interval shaded in gray, indicates a positive time-dependent trend [21,30,31,32,34,35,36,37,38,39,41,43,44,45,48,49,50,51,53,55].

Meta-regression analysis with respect to follow-up duration revealed a significant time-dependent effect only for LVEF. A longer follow-up period was associated with greater improvement in LVEF (*p* < 0.01), which may reflect a progressively increasing clinical benefit of physiological pacing in the context of left ventricular remodeling. This finding is illustrated in the bubble plot—Figure 20. For the remaining parameters—QRS duration, NYHA class, BNP, LVEDV, LVESV, R-wave amplitude, and pacing threshold—no significant time-related trends were identified. This may suggest that changes in these parameters stabilize within the first months after implantation, reaching a plateau effect.

#### 3.9.5. Summary of Publication Bias Across All Outcomes

Publication bias tests (Egger’s test and the trim-and-fill method) were conducted for the main parameters (LVEF, NYHA, BNP, QRS). In most analyses, no significant indications of publication bias were observed:

- Egger’s test: no funnel plot asymmetry detected for LVEF, BNP, QRS, or NYHA;
- Trim-and-fill: no imputed studies were added, supporting the robustness of the results.

For technical parameters (R-wave amplitude, pacing threshold), publication bias tests were not performed due to the small number of included studies (*k* < 10). Additionally, a Rosenthal Fail-Safe N (FSN) analysis was conducted (Table 8), which demonstrated that the results for LVEF and NYHA remain robust even in the presence of a large number of hypothetically unpublished studies. In contrast, the effect for LVEDV appeared to be more susceptible to potential file drawer bias.

## 4. Discussion

This meta-analysis, encompassing 8777 patients, demonstrates that CSP, including HBP and LBBAP, is associated with significant improvements in key functional and structural cardiac outcomes compared with conventional pacing strategies, including both RVP and BVP. CSP was linked to a significant increase in LVEF, a reduction in LVESV and LVEDV, as well as improvement in the NYHA functional class and a decrease in NT-proBNP levels, indicating favorable effects on both symptoms and cardiac remodeling. These benefits were consistently observed across subgroup and meta-regression analyses, supporting the physiological rationale and growing clinical relevance of CSP in patients requiring pacing therapy.

The most pronounced effect was observed with LBBAP, which is consistent with findings from a multicenter study showing its superiority over conventional BVP in terms of reverse remodeling, reduction in mortality, and HFH [55]. Wu et al. [53] compared LBBAP, HBP, and conventional BVP in patients with typical left bundle branch block (LBBB) and an LVEF ≤ 40%. At 12-month follow-up, both physiological pacing techniques demonstrated a comparable, greater increase in LVEF (about +24 percentage points) and normalization of LVEF, a significant QRS narrowing, and a greater improvement in the NYHA functional class compared with BVP. Additionally, LBBAP was characterized by a consistently low pacing threshold and higher R-wave amplitude, resulting in lower energy consumption and fewer reinterventions. Moreover, the composite endpoint of death or HFH occurred less frequently in the CSP group compared with the BVP group [56]. Similarly, in a study comparing left ventricular septal pacing (LVSP) with BVP and LBBAP, LBBAP demonstrated superior clinical outcomes compared with non-physiological pacing, while LVSP and BVP yielded comparable results to each other [57].

BVP is effective in patients with HF, bundle branch block (BBB), or those dependent on RVP. Currently, HBP is described as a rescue strategy in cases of BVP failure, although emerging evidence supports the feasibility and efficacy of HBP as a primary pacing strategy [43]. Although HBP is capable of correcting LBBB, its long-term use is limited by a higher risk of pacing threshold increase and technical challenges related to lead positioning [48]. LBBAP has a slight advantage over HBP, characterized by lower and more stable pacing thresholds in patients with LBBB and non-ischemic cardiomyopathy [38]. Wang et al. [51] demonstrated in a randomized trial involving patients with complete LBBB and non-ischemic cardiomyopathy that implantation of LBBAP for CRT resulted, after six months, in a 5.6-percentage-point greater increase in LVEF compared to conventional BVP, and a significantly greater reduction in LVESV (by 25 mL) and NT-proBNP (by 1.07 ng/mL), with comparable improvement in the NYHA functional class and similar QRS narrowing. Consistently low capture thresholds support long-term reliability and more favorable energy consumption.

The clinical benefits of CSP can be explained by the physiological activation of the myocardium through stimulation of the conduction system. Direct activation of the Purkinje network by CSP results in faster and more synchronized myocardial depolarization, which translates into improved systolic function and a reduction in left ventricular wall stress—the stretching force acting on the ventricular wall, responsible for pathological remodeling in HF. This approach eliminates electrical and mechanical dyssynchrony, prevents adverse left ventricular remodeling, and reduces the risk of HFH, atrial arrhythmias, and mortality induced by conventional RVP or BVP techniques. QRS narrowing further limits dyssynchrony and helps prevent pacing-induced cardiomyopathy observed with chronic RVP or BVP [58,59]. Moreover, in the absence of worsening cardiac function, CSP actively improves it. Meta-regression analysis demonstrated that the longer the follow-up period, the greater the increase in LVEF, suggesting a gradual and sustained process of reverse remodeling.

According to the current 2021 ESC guidelines, HBP may be considered for the following:

1. In candidates for CRT in whom implantation of a coronary sinus lead is unsuccessful (Class IIa recommendation);
2. With a backup ventricular lead in patients with rapidly conducted supraventricular arrhythmias undergoing a “pace-and-ablate” strategy (Class IIb recommendation);
3. As an alternative to RVP in patients with atrioventricular block (AVB) and an LVEF > 40%, who are expected to require >20% ventricular pacing (Class IIb recommendation).

Due to limited data, no official recommendations have been formulated regarding the use of LBBAP. Experts believe that this technique will play an increasingly important role in the future; however, the inclusion of LBBAP in official guidelines requires the publication of robust evidence confirming its safety and efficacy [60]. The authors of the 2023 HRS/APHRS/LAHRS guidelines introduced the concept of “cardiac physiologic pacing” (CPP) and indicate that CPP is recommended as follows:

1. In patients with an LVEF of 36–50% and an anticipated high percentage of RVP > 20–40% (Class I recommendation);
2. In patients with pacing-induced cardiomyopathy (PICM), defined as a decline in LVEF or the onset of HF symptoms associated with a high percentage of RVP (Class I recommendation).

CPP may be considered in patients with normal LVEF and an anticipated high percentage of RVP; in patients with an LVEF of 36–50% and a low percentage of RVP (<20–40%); in cases of unsuccessful CRT in patients with an LVEF ≤ 35%, sinus rhythm, LBBB, QRS duration ≥ 150 ms, and NYHA class II–IV; in patients with an LVEF of 36–50%, LBBB, QRS duration ≥ 150 ms, and NYHA class II–IV; and in patients undergoing AVJ ablation with an LVEF ≤ 50% [61].

Our results, similar to those of the Randomized Controlled Trial (RCT) conducted by Wang et al. [51] strengthen the rationale for upgrading the class of recommendations, particularly for LBBAP in candidates for CRT. Our data also expand upon previous reviews. Peng et al. [62] confirmed the superiority of CSP over RVP in improving cardiac function, implantation success rates, mean procedure time, fluoroscopy time, pacing thresholds, and reducing HFH; however, they did not evaluate BVP or biomarkers. Liu et al. [63] demonstrated a significant increase in LVEF, QRS narrowing, reduction in left ventricular end-diastolic diameter (LVEDD), improvement in the NYHA functional class, and significantly higher rates of echocardiographic and clinical response for LBBAP compared with BVP; however, their observation period was limited to 6–12 months. In contrast, we analyzed a population five times larger, with a longer follow-up (median 12 months), and additionally demonstrated a reduction in NT-proBNP levels. A decrease in NT-proBNP, a recognized prognostic biomarker in HF, suggests genuine hemodynamic improvement rather than merely an echocardiographic effect. Although echocardiographic parameters, clinical outcomes, and HF biomarkers provide valuable information on treatment efficacy, they cannot substitute for mortality data. Due to the lack of standardized data regarding procedural complications, implantation time, fluoroscopy duration, and technical success rates, our study did not analyze safety outcomes. Such data should be collected prospectively in future studies. Although CSP is not yet the standard approach in current ESC guidelines for most patients, our analysis provides strong evidence supporting the broader implementation of CSP, particularly in patients at high risk of HF.

The heterogeneity in our study (I^2^ > 90%) resulted from variability in follow-up duration (4–60 months), differences in implantation techniques (selective vs. non-selective capture), and the broad spectrum of patient populations (e.g., HF with or without AF, with or without LBBB, and various comorbidities). Stratification by implantation technique and time-dependent analysis partially reduced this variability; however, conclusions should be interpreted as applying to the average patient population rather than to individual cases. Although only a few studies were randomized, the direction of the effects we observed remains consistent with registry data, thereby strengthening the credibility of our findings.

Head-to-head comparisons further emphasized the superiority of CSP techniques over conventional approaches (RVP, BVP) in both electrophysiological and clinical parameters. It is important to highlight that improvements in the NYHA functional class and reductions in BNP levels were observed in parallel with echocardiographic improvements, strengthening the interpretation of the results as clinically meaningful rather than merely statistically significant.

In the future, large multicenter RCTs are needed to evaluate the efficacy and safety of HBP and LBBAP compared with conventional pacing methods over a long-term follow-up period (≥24 months). Meta-analyses also provide substantial value in the development of new guidelines, as they combine results from multiple studies, increase the statistical power of analyses, and allow for more precise estimation of the clinical effect of interventions. Meta-analyses represent the highest level of evidence in the hierarchy of Evidence-Based Medicine. We conducted our meta-analysis with the hope that it will provide reliable data and contribute to the further advancement of clinical practice and the development of future guidelines.

In conclusion, CSP provides consistent and clinically significant hemodynamic benefits compared with RVP and BVP. Although heterogeneity and the predominance of observational studies limit the certainty of the evidence, the consistency of findings across RCTs, registries, and the present meta-analysis highlights the real potential of this technology. Our results support the concept that CSP—particularly LBBAP—may become the preferred physiological pacing strategy in patients at risk of developing HF. Considering the observed benefits and current data limitations, there is an urgent need for large, prospective, multicenter RCTs comparing CSP with BiVP and RVP across different HF phenotypes.

## 5. Limitations, Strengths, and Future Directions

Despite the robust methodology and the number of comparisons included, this meta-analysis is subject to several limitations that should be considered when interpreting the results. The number of figures and tables was limited to the minimum necessary for a clear presentation of the findings. Duplicates and less relevant plots were removed, and each figure included presents a distinct parameter or analytical aspect, in line with PRISMA transparency guidelines. The vast majority of the included studies were observational in nature, which carries a risk of selection bias and confounding by indication. Application of the GRADE system [29] resulted in downgrading the certainty of evidence to a low level for all major outcomes, primarily reflecting the limited number of randomized trials and considerable inconsistency in effect sizes. Table 9 summarizes all identified limitations and outlines the strategies used by the authors to mitigate each one.

A major strength of the present study is its rigorous methodology, based on the PRISMA 2020 guidelines, including clearly defined inclusion and exclusion criteria, detailed methodological quality assessment using the QualSyst tool, and evaluation of publication bias through the trim-and-fill method and Egger’s test. The meta-analysis includes a large number of patients (a total of 8777 individuals), which increases the precision of clinical effect estimates and enhances the reliability of the presented results. The use of meta-regression and subgroup analyses enabled the identification of sources of heterogeneity, while the inclusion of both pre-post and head-to-head comparisons allowed for a better understanding of the differences between physiological and conventional pacing techniques. The presented findings provide practically relevant evidence supporting the benefits of physiological pacing techniques, which may directly influence clinical practice.

A limitation of our study is the inability to stratify outcomes based on conduction system capture status. Since confirmed capture strengthens the causal link between the pacing technique and its physiological effects, the lack of consistently reported data limits the depth of interpretation. Future studies should aim to report this aspect in a standardized manner to better define the effectiveness of CSP.

Moreover, an analysis of outcomes stratified by pacing indication (e.g., bradycardia vs. CRT) was not feasible due to the limited number of studies dedicated to specific indications. In particular, studies focusing exclusively on bradycardia were scarce and often evaluated multiple CSP techniques within small cohorts, precluding meaningful subgroup comparisons. Future meta-analyses may benefit from more uniform reporting and indication-specific data to clarify potential differential effects of CSP.

Another important limitation is that arrhythmic outcomes, such as ventricular arrhythmias, were not included in the present analysis due to insufficient and heterogeneous reporting across studies. As CSP may theoretically reduce dyssynchrony-related arrhythmogenesis, future prospective trials should assess its effect on arrhythmic burden using standardized definitions and reporting frameworks.

The results of the present study also highlight areas requiring further research. There is a need for RCTs directly comparing HBP and LBBAP techniques over longer follow-up periods, taking into account not only echocardiographic and electrophysiological parameters but also endpoints such as mortality and rehospitalization. Future studies should also focus on the standardization of implantation protocols and measurement methods, particularly regarding pacing selectivity and device parameters (pacing threshold, sensing, impedance). Further analyses should consider the use of individual patient data, which would allow for a more detailed assessment of the impact of potential effect moderators and help reduce the heterogeneity observed in the present work. Large multicenter RCTs with follow-up periods exceeding 24 months are necessary to verify the observed benefits under randomized conditions. These findings underscore the need for additional RCTs before any potential upgrade in guideline recommendation classes, given the currently low certainty of the available evidence.

## 6. Conclusions

CSP (HBP and LBBAP) provides a clearly greater hemodynamic benefit compared to conventional techniques (BVP and RVP), as evidenced by an increase in LVEF, a reduction in LVESV and LVEDV, an improvement in the NYHA class, and a decrease in NT-proBNP levels. The effect was consistent across subgroup analyses and head-to-head comparisons, and for LVEF, the benefit increased with longer follow-up durations (as shown by meta-regression). The results suggest the potential for an upgrade in the recommendation class, provided that these findings are confirmed by RCTs with hard clinical endpoints.

Ultimately, despite the high heterogeneity and the predominance of observational studies, our results support broader evaluation of CSP in well-designed RCTs with at least two years of follow-up and hard clinical endpoints.

## Figures and Tables

**Table 1:** PICO framework.

Population (P)	Patients with HF and various indications for pacemaker implantation.
Intervention (I)	Application of HBP or LBBAP.
Comparison (C)	Application of RVP or BVP.
Outcome (O)	LVEF, LVEDV, LVESV, QRS duration, NYHA, NT-proBNP, R-wave, pacing threshold.

Abbreviations: BVP, biventricular pacing; HBP, His bundle pacing; HF, heart failure; LBBAP, left bundle branch area pacing; LVEF, left ventricular ejection fraction; LVEDV, left ventricular end-diastolic volume; LVESV, left ventricular end-systolic volume; NT-proBNP, N-terminal pro–B-type natriuretic peptide; NYHA, New York Heart Association functional class; RVP, right ventricular pacing.

**Table 2:** The direction of SMD interpretation.

Variable	SMD < 0 Indicates	SMD > 0 Indicates	Clinical Benefit in the Analysis
LVEF	increase	decrease	SMD < 0
LVEDV, LVESV	increase	decrease	SMD > 0
NYHA	worsening	improvement	SMD > 0
NT-proBNP	increase	decrease	SMD > 0
QRS duration	extending	shortening	SMD > 0
Pacing threshold	decrease	increase	SMD < 0
R-wave amplitude	decrease	increase	SMD > 0

Abbreviations: LVEF, left ventricular ejection fraction; LVEDV, left ventricular end-diastolic volume; LVESV, left ventricular end-systolic volume; NT-proBNP, N-terminal pro–B-type natriuretic peptide; NYHA, New York Heart Association functional class; SMD, standardized mean difference.

**Table 3:** We present detailed data from each study.

Ref.	Authors (Year)	Total Patients	Summary QualSyst Score	Patient Population Characteristics	Intervention	Follow-Up [Months]
[15]	Abdelrahman et al. (2018)	765	0.79	Bradycardia	HBP, RVP	24.00
[30]	Chaumont et al. (2021)	170	0.71	General indications for pacing	HBP	11.00
[31]	Chaumont et al. (2024)	164	0.79	Arrhythmia with ANVA	HBP, LBBAP	12.00
[32]	Chen et al. (2022)	100	0.75	HF with LBBB, LVEF ≤ 35%	BVP, LBBAP	12.00
[33]	Chen et al. (2023)	1170	0.79	AVB	LBBAP	N/R
[34]	Chen et al. (2024)	145	0.75	CRT nonresponders (Sinus rhythm or AF)	LBBAP, BVP	24.00
[35]	Ezzeddine et al. (2023)	238	0.75	CRT nonresponders, HFrEF	BVP, CSP (HBP, LBBAP)	9.00
[36]	Guo et al. (2020)	42	0.75	HF with LBBB, LVEF ≤ 35%, NYHA II-IV	LBBAP, BVP	6.00
[37]	Huang et al. (2019)	74	0.71	HF with LBBB	HBP, BVP	12.00
[38]	Huang et al. (2020)	63	0.75	HF with LBBB, LVEF < 50%, nonischemic cardiomyopathy	LBBAP	18.00
[39]	Ponnusamy et al. (2021)	11	0.68	Octogenarians (≥80 years), with AVB, LBBB with low EF, SND.	LBBAP	7.70
[40]	Sharma et al. (2022)	50	0.71	Bradycardia	RVP	22.10
		703	0.79		LBBAP	16.20
[41]	Sefton et al. (2024)	27	0.71	AF and advanced pulmonary disease, AVNA	LBBAP	17.30
[42]	Sharma et al. (2015)	192	0.75	Bradycardia	HBP, RVP	25.50
[43]	Sharma, Dandamudi, et al. (2018)	106	0.75	HF and AVB, BBB and high rates of ventricular pacing	HBP	14.40
[44]	Sharma, Naperkowski, et al. (2018)	39	0.71	HF with RBBB, LVEF ≤ 50%, NYHA II-IV	HBP	15.00
[45]	Tang et al. (2025)	36	0.71	HF, LVEF 35–50%	BVP, CSP (HBP, LBBAP)	47.00
[46]	Upadhyay et al. (2019)	41	0.89	HF with LBBB/RBBB, NYHA II–IV	HBP, BVP	6.20
[47]	Vazquez et al. (2023)	849	0.75	AVB, HF with wide QRS, HF with frequent dependence on ventricular pacing, AVNA, Ischemic/nonischemic cardiomyopathy	HBP, LBBAP	5.00
[21]	Vijayaraman et al. (2018)	192	0.75	Bradycardia	HBP, RVP	60.00
[48]	Vijayaraman et al. (2021)	325	0.79	HF with LBBB, LVEF ≤ 50%, NYHA II-IV	LBBAP	6.00
[49]	Vijayaraman et al. (2023)	1778	0.79	CRT nonresponders, HFrEF, LVEF ≤ 35%	BVP, LBBAP	33.00
[50]	Vinther et al. (2021)	50	0.89	HF with LBBB, LVEF ≤ 35%, NYHA II-IV	HBP, BVP	6.00
[51]	Wang et al. (2022)	40	0.79	HF with LBBB, LVEF ≤ 40%, nonischemic cardiomyopathy	BVP, LBBAP	6.00
[52]	Whinnett et al. (2023)	167	1.00	HF, LVEF ≤ 40%, AVB 1.degree, QRS ≤ 140 ms or RBBB	HBP	6.00
[53]	Wu et al. (2021)	137	0.75	HF with LBBB, LVEF ≤ 40%	HBP, LBBAP, BVP	12.00
[54]	Zanon et al. (2019)	844	0.75	AVB, SND, bradycardia with AF, HF	HBP	3.90
[55]	Zhu et al. (2024)	259	0.79	HF, AVB, LBBB, RBBB, AF, LVEF < 50%	BVP, LBBAP	29.00

Abbreviations: AVB, atrioventricular block; AVNA, atrioventricular node ablation; BVP, biventricular pacing; CRT, cardiac resynchronization therapy; CSP, conduction system pacing; EF, ejection fraction; HBP, His bundle pacing; HF, heart failure; HFrEF, heart failure with reduced ejection fraction; LBBB, left bundle branch block; LBBAP, left bundle branch area pacing; LVEF, left ventricular ejection fraction; N/R, not reported; NYHA, New York Heart Association functional class; RBBB, right bundle branch block; RVP, right ventricular pacing; SND, sinus node dysfunction.

**Table 4:** Summary of findings (GRADE—key endpoints) [29].

Endpoint	Comparisons/Patients	Pooled Effect (SMD [95% CI])	Certainty of Evidence (GRADE)	Downgrading Factors
LVEF (benefit SMD < 0)	34/6102	−1.38 [−1.69; −1.06]	⬤⬤◯◯ low	observational studies, I^2^ > 90%
NYHA (benefit SMD > 0)	19/3418	1.94 [1.59; 2.29]	⬤⬤◯◯ low	observational studies, I^2^ > 90%
NT-proBNP (benefit SMD > 0)	5/811	0.91 [0.63; 1.19]	⬤⬤◯◯ low	a limited number of studies, observational studies
LVESV (benefit SMD > 0)	13/2047	1.14 [0.81; 1.47]	⬤⬤◯◯ low	heterogeneity, lack of Randomized Controlled Trials
Pacing threshold (benefit SMD < 0)	17/2682	−0.14 [−0.36; 0.08]	⬤⬤◯◯ low	non-significant effect, I^2^ = 81%

Legend: GRADE: ⬤⬤⬤⬤ high|⬤⬤⬤◯ moderate|⬤⬤◯◯ low|⬤◯◯◯ very low. Abbreviations: LVEF, left ventricular ejection fraction; LVESV, left ventricular end-systolic volume; NT-proBNP, N-terminal pro–B-type natriuretic peptide; NYHA, New York Heart Association functional class; SMD, standardized mean difference.

**Table 5:** Head-to-head effects (multi-arm studies only, random-effects model).

Parameter	SMD (95%CI)	*p*-Value	Interpretation
LVEF	−1.49 [−1.85; −1.13]	<0.0001	Significant improvement following physiological pacing
QRS duration	0.18 [−0.42; 0.79]	0.55	No difference observed
NYHA	1.94 [1.43; 2.45]	<0.0001	Significant improvement
NT-proBNP	0.92 [0.62; 1.22]	<0.0001	Significant improvement.
LVEDV	−0.23 [−0.44; −0.03]	0.025	Moderate improvement
LVESV	−0.35 [−0.60; −0.09]	0.0078	Improvement
Pacing threshold	−0.23 [−0.44; −0.03]	0.025	Potential benefit of physiological pacing
R-wave amplitude	−0.09 [−0.21; 0.03]	0.13	No significant difference

Abbreviations: CI, Confidence Interval; LVEF, left ventricular ejection fraction; LVEDV, left ventricular end-diastolic volume; LVESV, left ventricular end-systolic volume; NT-proBNP, N-terminal pro–B-type natriuretic peptide; NYHA, New York Heart Association functional class; SMD, standardized mean difference.

**Table 6:** Summary of meta-analysis results.

Parameter	Overall Effect (SMD [CI])	Overall *p*-Value	CSP vs. Conventional (*p*)	Meta-Regression (*p*)	Trend vs. Follow-Up
LVEF	−1.49 [−1.85, −1.13]	<0.0001	0.987	<0.01	Yes
QRS duration	0.18 [−0.42, 0.79]	0.55	0.333	0.134	No
NYHA	1.94 [1.43, 2.45]	<0.0001	0.286	0.134	No
NT-proBNP	0.92 [0.62, 1.22]	<0.0001	0.891	0.445	No
LVEDV	−0.23 [−0.44, −0.03]	0.025	0.134	0.134	No
LVESV	−0.35 [−0.60, −0.09]	0.0078	0.134	0.134	No
R-wave amplitude	−0.09 [−0.21, 0.03]	0.13	0.445	0.445	No
Pacing threshold	−0.23 [−0.44, −0.03]	0.025	0.134	0.134	No

The moderation *p*-value refers to the test of differences between CSP and conventional groups; “trend” indicates a significant association with follow-up duration in the meta-regression. Abbreviations: CI, Confidence Interval; CSP, conduction system pacing; LVEF, left ventricular ejection fraction; LVEDV, left ventricular end-diastolic volume; LVESV, left ventricular end-systolic volume; NT-proBNP, N-terminal pro–B-type natriuretic peptide; NYHA, New York Heart Association functional class; SMD, standardized mean difference.

**Table 7:** Pooled results of meta-regressions: impact of follow-up duration on effect size (SMD). To evaluate whether follow-up length influenced the strength of the effect, separate meta-regressions were performed for each parameter. A significant effect was observed only for LVEF.

Parameter	Number of Studies (*k*)	β (Regression Coefficient)	95%CI β	*p* for Follow-Up	Effect of Follow-Up
LVEF	24	+0.026	[0.011, 0.041]	<0.01	Significant
QRS duration	24	−0.007	[−0.022, 0.008]	0.13	Not significant
NYHA	15	−0.013	[−0.038, 0.011]	0.13	Not significant
NT-proBNP	4	−0.004	[−0.062, 0.054]	0.89	Not significant
LVEDV	8	+0.007	[−0.006, 0.021]	0.45	Not significant
LVESV	11	+0.013	[−0.004, 0.031]	0.13	Not significant
R-wave amplitude	9	−0.005	[−0.018, 0.008]	0.45	Not significant
Pacing threshold	11	+0.012	[−0.009, 0.033]	0.13	Not significant

β-value of the linear regression coefficient for the follow-up variable (in months), calculated using the REML method. Abbreviations: CI, Confidence Interval; LVEF, left ventricular ejection fraction; LVEDV, left ventricular end-diastolic volume; LVESV, left ventricular end-systolic volume; NT-proBNP, N-terminal pro–B-type natriuretic peptide; NYHA, New York Heart Association functional class.

**Table 8:** Summary of Rosenthal FSN analysis.

Parameter	*k*	SMD (95% CI)	Rosenthal Fail-Safe N	*p* After FSN Adjustment	Interpretation
LVEF	34	−1.38 (−1.69; −1.06)	512	0.0501	highly robust effect
NYHA	19	1.94 (1.59; 2.29)	410	0.0503	robust
LVESV	13	1.14 (0.81; 1.47)	95	0.0506	moderate robustness
LVEDV	9	0.66 (0.25; 1.08)	11	0.0574	sensitive to publication bias

The legend: Fail-Safe N (FSN) = number of hypothetical null-effect studies that would need to be added to lose significance at the α level = 0.05. Abbreviations: CI, Confidence Interval; FSN, Rosenthal Fail-Safe N; LVEF, left ventricular ejection fraction; LVEDV, left ventricular end-diastolic volume; LVESV, left ventricular end-systolic volume; NYHA, New York Heart Association functional class; SMD, standardized mean difference.

**Table 9:** Summary of meta-analysis limitations with justifications.

Parameter/Aspect	Limitation	Comment
general	heterogeneity of the results (high I^2^ values)	Expected due to population, methodological, and temporal differences; random-effects models, subgroup analyses, and meta-regressions with respect to follow-up were applied.
	predominance of observational studies	Most of the data originate from non-randomized studies; potential selection bias was minimized through head-to-head analyses and a rigorous assessment of methodological quality (QualSyst).
	lack of complete individual patient data	A standard limitation of meta-analyses; the best available aggregated data were used; the results should be confirmed in future individual patient data analyses.
	variable follow-up duration (3.9–60 months)	Meta-regression with respect to follow-up duration was applied to assess the impact of time on the outcomes.
	potential for publication bias	Assessment performed using Egger’s test and the trim-and-fill method; results were adjusted where necessary.
	lack of a standardized definition of CSP	Differences were accounted for in subgroup analyses; results were interpreted with caution.
	diversity of implantation protocols (e.g., selective vs. non-selective)	Results were interpreted with caution; detailed protocols should be standardized in future studies.
LVEF	lack of full standardization of assessment methods	Use of SMD; echocardiography was the predominant assessment method, adjusted using the REML model.
	lack of analysis of mortality and rehospitalization	Consistent with the PICO framework of the study (focused on LVEF); clinical outcomes should be addressed in separate investigations.
	potential overlap of patient populations	A mixed-effects analysis with clustering by publication number was applied.
LVEDV and LVESV	small number of studies in certain groups	Results were considered exploratory, with recommendations for further research.
	lack of raw data for delta analysis	Use of aggregated data, with SMD partially minimizing this limitation.
	use of SMD instead of mean differences	Justified by heterogeneity in measurement units and populations; the analysis is standardized.
QRS	possible multiple arms within studies	Separate head-to-head analysis for multi-arm studies; clearly discussed in the text.
NYHA	lack of randomized head-to-head studies comparing HBP and LBBAP	The need for future RCTs was emphasized; results were interpreted with caution.
NT-proBNP	small number of studies (*k* = 5)	Results were interpreted with caution; further studies with larger sample sizes are recommended.
	lack of long-term data	Meta-regression did not demonstrate an impact of follow-up duration; however, the results require confirmation in longer-term observations.
R-Wave Amplitude	heterogeneity of measurement methods	Clear indication of the lack of standardization in the original studies; results were interpreted as preliminary.
	lack of data on device settings (gain, sensing)	Potential source of technical bias; recommendation for future standardization in clinical studies.
Pacing Threshold	non-random selection of studies	Only complete analyses (Means, Standard Deviation, N) were used; the risk of publication bias is low.
	lack of data on secondary outcomes	The need for future studies focusing on the technical parameters of the leads was indicated.
	small number of comparisons between HBP and LBBAP	Results were presented with caution, emphasizing the need for further studies.

Abbreviations: HBP, His bundle pacing; LBBAP, left bundle branch area pacing; LVEF, left ventricular ejection fraction; LVEDV, left ventricular end-diastolic volume; LVESV, left ventricular end-systolic volume; NT-proBNP, N-terminal pro–B-type natriuretic peptide; NYHA, New York Heart Association functional class; RCT, Randomized Controlled Trial; SMD, standardized mean difference.

## Data Availability

This study is based on previously published data. No new data were generated or analyzed.

## Supplementary Materials

The following supporting information can be downloaded at https://www.mdpi.com/article/10.3390/biomedicines13061359/s1. Supplementary File S1 includes additional figures referenced in the manuscript. Figure S1: Forest plot for the overall meta-analysis of the effect of cardiac pacing on left ventricular ejection fraction (LVEF); Figure S2: Forest plot for the overall meta-analysis of the effect of cardiac pacing on left ventricular end-diastolic volume (LVEDV); Figure S3: Forest plot for the overall meta-analysis of the effect of cardiac pacing on left ventricular end-systolic volume (LVESV); Figure S4: Forest plot for the overall meta-analysis of the effect of cardiac pacing on QRS duration; Figure S5: Forest plot for the overall meta-analysis of the effect of cardiac pacing on NYHA class; Figure S6: Forest plot for the overall meta-analysis of the effect of cardiac pacing on BNP level; Figure S7: Forest plot for the overall meta-analysis of the effect of cardiac pacing on R-wave amplitude; Figure S8: Forest plot for the overall meta-analysis of the effect of cardiac pacing on pacing threshold.

## Abbreviations

The following abbreviations are used in this manuscript:

AF	atrial fibrillation
AVB	atrioventricular block
AVJ	atrioventricular junction
AVNA	atrioventricular node ablation
BNP	Brain Natriuretic Peptide
BVP	biventricular pacing
CI	Confidence Interval
CSP	conduction system pacing
CRT	cardiac resynchronization therapy
EF	ejection fraction
FSN	Fail-Safe N
HBP	His bundle pacing
HF	heart failure
HFH	heart failure hospitalization
HFrEF	heart failure with reduced ejection fraction
LBBAP	left bundle branch area pacing
LBBB	left bundle branch block
LVEDD	left ventricular end-diastolic diameter
LVEDV	left ventricular end-diastolic volume
LVEF	left ventricular ejection fraction
LVESV	left ventricular end-systolic volume
NMA	network meta-analysis
NT-proBNP	N-terminal pro–B-type natriuretic peptide
NYHA	New York Heart Association functional class
PICM	pacemaker-induced cardiomyopathy
RBBB	right bundle branch block
RCT	Randomized Controlled Trial
RVP	right ventricular pacing
SE	Standard Errors
SMD	standardized mean difference
SND	sinus node dysfunction

## References

[1] Aktaa S., Abdin A., Arbelo E., Burri H., Vernooy K., Blomström-Lundqvist C., Boriani G., Defaye P., Deharo J.-C., Drossart I. (2022). European Society of Cardiology Quality Indicators for the Care and Outcomes of Cardiac Pacing: Developed by the Working Group for Cardiac Pacing Quality Indicators in Collaboration with the European Heart Rhythm Association of the European Society of Cardiology. EP Eur..

[2] Oida M., Mizutani T., Hasumi E., Fujiu K., Goto K., Kani K., Oshima T., Matsubara T.J., Shimizu Y., Oguri G. (2024). Prediction of Pacemaker-Induced Cardiomyopathy Using a Convolutional Neural Network Based on Clinical Findings Prior to Pacemaker Implantation. Sci. Rep..

[3] Yu C., Fang F., Luo X., Zhang Q., Azlan H., Razali O. (2014). Long-term Follow-up Results of the Pacing to Avoid Cardiac Enlargement (PACE) Trial. Eur. J. Heart Fail..

[4] Moss A.J., Hall W.J., Cannom D.S., Klein H., Brown M.W., Daubert J.P., Estes N.A.M., Foster E., Greenberg H., Higgins S.L. (2009). Cardiac-Resynchronization Therapy for the Prevention of Heart-Failure Events. N. Engl. J. Med..

[5] Tang A.S.L., Wells G.A., Talajic M., Arnold M.O., Sheldon R., Connolly S., Hohnloser S.H., Nichol G., Birnie D.H., Sapp J.L. (2010). Cardiac-Resynchronization Therapy for Mild-to-Moderate Heart Failure. N. Engl. J. Med..

[6] Curtis A.B., Worley S.J., Adamson P.B., Chung E.S., Niazi I., Sherfesee L., Shinn T., St. John Sutton M. (2013). Biventricular Pacing for Atrioventricular Block and Systolic Dysfunction. N. Engl. J. Med..

[7] Cleland J.G.F., Daubert J.-C., Erdmann E., Freemantle N., Gras D., Kappenberger L., Tavazzi L. (2005). The Effect of Cardiac Resynchronization on Morbidity and Mortality in Heart Failure. N. Engl. J. Med..

[8] Bristow M.R., Saxon L.A., Boehmer J., Krueger S., Kass D.A., De Marco T., Carson P., DiCarlo L., DeMets D., White B.G. (2004). Cardiac-Resynchronization Therapy with or without an Implantable Defibrillator in Advanced Chronic Heart Failure. N. Engl. J. Med..

[9] Naqvi S.Y., Jawaid A., Goldenberg I., Kutyifa V. (2018). Non-Response to Cardiac Resynchronization Therapy. Curr. Heart Fail. Rep..

[10] Chairs T.F., Daubert J.-C., Saxon L., Adamson P.B., Auricchio A., Berger R.D., Beshai J.F., Breithard O., Brignole M., Cleland J. (2012). 2012 EHRA/HRS Expert Consensus Statement on Cardiac Resynchronization Therapy in Heart Failure: Implant and Follow-up Recommendations and Management: A Registered Branch of the European Society of Cardiology (ESC), and the Heart Rhythm Society; and in Collaboration with the Heart Failure Society of America (HFSA), the American Society of Echocardiography (ASE), the American Heart Association (AHA), the European Association of Echocardiography (EAE) of the ESC and the Heart Failure Association of the ESC (HFA). * Endorsed by the Governing Bodies of AHA, ASE, EAE, HFSA, HFA, EHRA, and HRS. Europace.

[11] Prinzen F.W., Vernooy K., Auricchio A. (2013). Cardiac Resynchronization Therapy: State-of-the-Art of Current Applications, Guidelines, Ongoing Trials, and Areas of Controversy. Circulation.

[12] Scherlag B.J., Kosowsky B.D., Damato A.N. (1967). A Technique for Ventricular Pacing from the His Bundle of the Intact Heart. J. Appl. Physiol..

[13] Deshmukh P., Casavant D.A., Romanyshyn M., Anderson K. (2000). Permanent, Direct His-Bundle Pacing: A Novel Approach to Cardiac Pacing in Patients With Normal His-Purkinje Activation. Circulation.

[14] Catanzariti D., Maines M., Cemin C., Broso G., Marotta T., Vergara G. (2006). Permanent Direct His Bundle Pacing Does Not Induce Ventricular Dyssynchrony Unlike Conventional Right Ventricular Apical Pacing: An Intrapatient Acute Comparison Study. J. Interv. Card. Electrophysiol..

[15] Abdelrahman M., Subzposh F.A., Beer D., Durr B., Naperkowski A., Sun H., Oren J.W., Dandamudi G., Vijayaraman P. (2018). Clinical Outcomes of His Bundle Pacing Compared to Right Ventricular Pacing. J. Am. Coll. Cardiol..

[16] Huang W., Su L., Wu S., Xu L., Xiao F., Zhou X., Ellenbogen K.A. (2017). A Novel Pacing Strategy With Low and Stable Output: Pacing the Left Bundle Branch Immediately Beyond the Conduction Block. Can. J. Cardiol..

[17] Zhuo W., Zhong X., Liu H., Yu J., Chen Q., Hu J., Xiong Q., Hong K. (2022). Pacing Characteristics of His Bundle Pacing vs. Left Bundle Branch Pacing: A Systematic Review and Meta-Analysis. Front. Cardiovasc. Med..

[18] Prinzen F.W., Hunter W.C., Wyman B.T., McVeigh E.R. (1999). Mapping of Regional Myocardial Strain and Work during Ventricular Pacing: Experimental Study Using Magnetic Resonance Imaging Tagging. J. Am. Coll. Cardiol..

[19] Mariani M.V., Piro A., Forleo G.B., Della Rocca D.G., Natale A., Miraldi F., Vizza C.D., Lavalle C. (2023). Clinical, Procedural and Lead Outcomes Associated with Different Pacing Techniques: A Network Meta-Analysis. Int. J. Cardiol..

[20] Herweg B., Sharma P.S., Cano Ó., Ponnusamy S.S., Zanon F., Jastrzebski M., Zou J., Chelu M.G., Vernooy K., Whinnett Z.I. (2024). Arrhythmic Risk in Biventricular Pacing Compared With Left Bundle Branch Area Pacing: Results From the I-CLAS Study. Circulation.

[21] Vijayaraman P., Naperkowski A., Subzposh F.A., Abdelrahman M., Sharma P.S., Oren J.W., Dandamudi G., Ellenbogen K.A. (2018). Permanent His-Bundle Pacing: Long-Term Lead Performance and Clinical Outcomes. Heart Rhythm.

[22] Mirmaksudov M., Ross S., Kongsgård E., Edvardsen T. (2024). Enhancing Cardiac Pacing Strategies: A Review of Conduction System Pacing Compared with Right and Biventricular Pacing and Their Influence on Myocardial Function. Eur. Heart J.-Cardiovasc. Imaging.

[23] Hua J., Wang C., Kong Q., Zhang Y., Wang Q., Xiong Z., Hu J., Li J., Chen Q., Hong K. (2022). Comparative Effects of Left Bundle Branch Area Pacing, His Bundle Pacing, Biventricular Pacing in Patients Requiring Cardiac Resynchronization Therapy: A Network Meta-analysis. Clin. Cardiol..

[24] Domenichini G., Le Bloa M., Teres Castillo C., Graf D., Carroz P., Ascione C., Porretta A.P., Pascale P., Pruvot E. (2023). Conduction System Pacing versus Conventional Biventricular Pacing for Cardiac Resynchronization Therapy: Where Are We Heading?. J. Clin. Med..

[25] Page M.J., McKenzie J.E., Bossuyt P.M., Boutron I., Hoffmann T.C., Mulrow C.D., Shamseer L., Tetzlaff J.M., Akl E.A., Brennan S.E. (2021). The PRISMA 2020 Statement: An Updated Guideline for Reporting Systematic Reviews. BMJ.

[26] Haddaway N.R., Page M.J., Pritchard C.C., McGuinness L.A. (2022). *PRISMA2020*: An R Package and Shiny App for Producing PRISMA 2020-compliant Flow Diagrams, with Interactivity for Optimised Digital Transparency and Open Synthesis. Campbell Syst. Rev..

[27] Kmet L.M., Cook L.S., Lee R.C. Standard Quality Assessment Criteria for Evaluating Primary Research Papers from a Variety of Fields 2004. https://era.library.ualberta.ca/items/48b9b989-c221-4df6-9e35-af782082280e/view/a1cffdde-243e-41c3-be98-885f6d4dcb29/standard_quality_assessment_criteria_for_evaluating_primary_research_papers_from_a_variety_of_fields.pdf.

[28] Higgins J., Thomas J., Chandler J., Cumpston M., Li T., Page M., Velch V. Cochrane Handbook for Systematic Reviews of Interventions Version 6.5 (Updated August 2024). https://training.cochrane.org/handbook.

[29] Akl E., Mustafa R., Wiercioch N., Schünemann H., Brożek J., Guyatt G. (2013). GRADE Handbook.

[30] Chaumont C., Auquier N., Milhem A., Mirolo A., Al Arnaout A., Popescu E., Viart G., Godin B., Gillibert A., Savouré A. (2021). Can Permanent His Bundle Pacing Be Safely Started by Operators New to This Technique? Data from a Multicenter Registry. Cardiovasc. Electrophysiol..

[31] Chaumont C., Azincot M., Savouré A., Auquier N., Hamoud R.A., Popescu E., Viart G., Mirolo A., Eltchaninoff H., Anselme F. (2024). His Bundle Pacing versus Left Bundle Branch Area Pacing in Patients Undergoing Atrioventricular Node Ablation: A Prospective and Comparative Study. Arch. Cardiovasc. Dis..

[32] Chen X., Ye Y., Wang Z., Jin Q., Qiu Z., Wang J., Qin S., Bai J., Wang W., Liang Y. (2022). Cardiac Resynchronization Therapy via Left Bundle Branch Pacing vs. Optimized Biventricular Pacing with Adaptive Algorithm in Heart Failure with Left Bundle Branch Block: A Prospective, Multi-Centre, Observational Study. EP Eur..

[33] Chen Z., Xu Y., Jiang L., Zhang R., Zhao H., Liu R., Zhang L., Li Y., Liu X. (2023). Left Bundle Branch Area Pacing versus Right Ventricular Pacing in Patients with Atrioventricular Block: An Observational Cohort Study. Cardiovasc. Ther..

[34] Chen X., Jin Q., Qiu Z., Qian C., Liang Y., Wang J., Qin S., Bai J., Wang W., Chen H. (2024). Outcomes of Upgrading to LBBP in CRT Nonresponders. JACC Clin. Electrophysiol..

[35] Ezzeddine F.M., Pistiolis S.M., Pujol-Lopez M., Lavelle M., Wan E.Y., Patton K.K., Robinson M., Lador A., Tamirisa K., Karim S. (2023). Outcomes of Conduction System Pacing for Cardiac Resynchronization Therapy in Patients with Heart Failure: A Multicenter Experience. Heart Rhythm.

[36] Guo J., Li L., Xiao G., Ye T., Huang X., Meng F., Li Q., Chen S., Cai B. (2020). Remarkable Response to Cardiac Resynchronization Therapy via Left Bundle Branch Pacing in Patients with True Left Bundle Branch Block. Clin. Cardiol..

[37] Huang W., Su L., Wu S., Xu L., Xiao F., Zhou X., Mao G., Vijayaraman P., Ellenbogen K.A. (2019). Long-Term Outcomes of His Bundle Pacing in Patients with Heart Failure with Left Bundle Branch Block. Heart.

[38] Huang W., Wu S., Vijayaraman P., Su L., Chen X., Cai B., Zou J., Lan R., Fu G., Mao G. (2020). Cardiac Resynchronization Therapy in Patients With Nonischemic Cardiomyopathy Using Left Bundle Branch Pacing. JACC Clin. Electrophysiol..

[39] Ponnusamy S.S., Bopanna D., Syed T., Muthu G., Kumar S. (2021). Feasibility, Safety and Outcomes of Left Bundle Branch Pacing in Octogenarians. Indian Heart J..

[40] Sharma P.S., Patel N.R., Ravi V., Zalavadia D.V., Dommaraju S., Garg V., Larsen T.R., Naperkowski A.M., Wasserlauf J., Krishnan K. (2022). Clinical Outcomes of Left Bundle Branch Area Pacing Compared to Right Ventricular Pacing: Results from the Geisinger-Rush Conduction System Pacing Registry. Heart Rhythm.

[41] Sefton C., Tanaka-Esposito C., Dresing T., Lee J., Chung R. (2024). Outcomes of combined left bundle branch area pacing with atrioventricular nodal ablation in patients with atrial fibrillation and pulmonary disease. Pacing Clin. Electrophis.

[42] Sharma P.S., Dandamudi G., Naperkowski A., Oren J.W., Storm R.H., Ellenbogen K.A., Vijayaraman P. (2015). Permanent His-Bundle Pacing Is Feasible, Safe, and Superior to Right Ventricular Pacing in Routine Clinical Practice. Heart Rhythm.

[43] Sharma P.S., Dandamudi G., Herweg B., Wilson D., Singh R., Naperkowski A., Koneru J.N., Ellenbogen K.A., Vijayaraman P. (2018). Permanent His-Bundle Pacing as an Alternative to Biventricular Pacing for Cardiac Resynchronization Therapy: A Multicenter Experience. Heart Rhythm.

[44] Sharma P.S., Naperkowski A., Bauch T.D., Chan J.Y.S., Arnold A.D., Whinnett Z.I., Ellenbogen K.A., Vijayaraman P. (2018). Permanent His Bundle Pacing for Cardiac Resynchronization Therapy in Patients With Heart Failure and Right Bundle Branch Block. Circ. Arrhythmia Electrophysiol..

[45] Tang J., Kong N.W., Beaser A., Aziz Z., Yeshwant S., Ozcan C., Tung R., Upadhyay G.A. (2025). Clinical Outcomes of Conduction System Pacing Compared to Biventricular Pacing in Patients with Mid-Range Ejection Fraction. J. Interv. Card. Electrophysiol..

[46] Upadhyay G.A., Vijayaraman P., Nayak H.M., Verma N., Dandamudi G., Sharma P.S., Saleem M., Mandrola J., Genovese D., Oren J.W. (2019). On-Treatment Comparison between Corrective His Bundle Pacing and Biventricular Pacing for Cardiac Resynchronization: A Secondary Analysis of the His-SYNC Pilot Trial. Heart Rhythm.

[47] Vazquez P.M., Mohamed U., Zanon F., Lustgarten D.L., Atwater B., Whinnett Z.I., Curila K., Dinerman J., Molina-Lerma M., Wiley J. (2023). Result of the Physiologic Pacing Registry, an International Multicenter Prospective Observational Study of Conduction System Pacing. Heart Rhythm.

[48] Vijayaraman P., Ponnusamy S., Cano Ó., Sharma P.S., Naperkowski A., Subsposh F.A., Moskal P., Bednarek A., Dal Forno A.R., Young W. (2021). Left Bundle Branch Area Pacing for Cardiac Resynchronization Therapy. JACC Clin. Electrophysiol..

[49] Vijayaraman P., Sharma P.S., Cano Ó., Ponnusamy S.S., Herweg B., Zanon F., Jastrzebski M., Zou J., Chelu M.G., Vernooy K. (2023). Comparison of Left Bundle Branch Area Pacing and Biventricular Pacing in Candidates for Resynchronization Therapy. J. Am. Coll. Cardiol..

[50] Vinther M., Risum N., Svendsen J.H., Møgelvang R., Philbert B.T. (2021). A Randomized Trial of His Pacing Versus Biventricular Pacing in Symptomatic HF Patients With Left Bundle Branch Block (His-Alternative). JACC Clin. Electrophysiol..

[51] Wang Y., Zhu H., Hou X., Wang Z., Zou F., Qian Z., Wei Y., Wang X., Zhang L., Li X. (2022). Randomized Trial of Left Bundle Branch vs Biventricular Pacing for Cardiac Resynchronization Therapy. J. Am. Coll. Cardiol..

[52] Whinnett Z.I., Shun-Shin M.J., Tanner M., Foley P., Chandrasekaran B., Moore P., Adhya S., Qureshi N., Muthumala A., Lane R. (2023). Effects of Haemodynamically Atrio-ventricular Optimized His Bundle Pacing on Heart Failure Symptoms and Exercise Capacity: The His Optimized Pacing Evaluated for Heart Failure (HOPE-HF) Randomized, Double-blind, Cross-over Trial. Eur. J. Heart Fail..

[53] Wu S., Su L., Vijayaraman P., Zheng R., Cai M., Xu L., Shi R., Huang Z., Whinnett Z.I., Huang W. (2021). Left Bundle Branch Pacing for Cardiac Resynchronization Therapy: Nonrandomized On-Treatment Comparison With His Bundle Pacing and Biventricular Pacing. Can. J. Cardiol..

[54] Zanon F., Abdelrahman M., Marcantoni L., Naperkowski A., Subzposh F.A., Pastore G., Baracca E., Boaretto G., Raffagnato P., Tiribello A. (2019). Long Term Performance and Safety of His Bundle Pacing: A Multicenter Experience. Cardiovasc. Electrophysiol..

[55] Zhu H., Qin C., Du A., Wang Q., He C., Zou F., Li X., Tao J., Wang C., Liu Z. (2024). Comparisons of Long-Term Clinical Outcomes with Left Bundle Branch Pacing, Left Ventricular Septal Pacing, and Biventricular Pacing for Cardiac Resynchronization Therapy. Heart Rhythm.

[56] Vijayaraman P., Zalavadia D., Haseeb A., Dye C., Madan N., Skeete J.R., Vipparthy S.C., Young W., Ravi V., Rajakumar C. (2022). Clinical Outcomes of Conduction System Pacing Compared to Biventricular Pacing in Patients Requiring Cardiac Resynchronization Therapy. Heart Rhythm.

[57] Diaz J.C., Tedrow U.B., Duque M., Aristizabal J., Braunstein E.D., Marin J., Niño C., Bastidas O., Lopez Cabanillas N., Koplan B.A. (2024). Left Bundle Branch Pacing vs Left Ventricular Septal Pacing vs Biventricular Pacing for Cardiac Resynchronization Therapy. JACC Clin. Electrophysiol..

[58] Ponnusamy S.S., Syed T., Vijayaraman P. (2023). Pacing Induced Cardiomyopathy: Recognition and Management. Heart.

[59] Somma V., Ha F.J., Palmer S., Mohamed U., Agarwal S. (2023). Pacing-Induced Cardiomyopathy: A Systematic Review and Meta-Analysis of Definition, Prevalence, Risk Factors, and Management. Heart Rhythm.

[60] Glikson M., Nielsen J.C., Kronborg M.B., Michowitz Y., Auricchio A., Barbash I.M., Barrabés J.A., Boriani G., Braunschweig F., Brignole M. (2021). 2021 ESC Guidelines on Cardiac Pacing and Cardiac Resynchronization Therapy. Eur. Heart J..

[61] Chung M.K., Patton K.K., Lau C.-P., Dal Forno A.R.J., Al-Khatib S.M., Arora V., Birgersdotter-Green U.M., Cha Y.-M., Chung E.H., Cronin E.M. (2023). 2023 HRS/APHRS/LAHRS Guideline on Cardiac Physiologic Pacing for the Avoidance and Mitigation of Heart Failure. Heart Rhythm.

[62] Peng X., Chen Y., Wang X., Hu A., Li X. (2021). Safety and Efficacy of His-Bundle Pacing/Left Bundle Branch Area Pacing versus Right Ventricular Pacing: A Systematic Review and Meta-Analysis. J. Interv. Card. Electrophysiol..

[63] Liu J., Sun F., Wang Z., Sun J., Jiang X., Zhao W., Zhang Z., Liu L., Zhang S. (2021). Left Bundle Branch Area Pacing vs. Biventricular Pacing for Cardiac Resynchronization Therapy: A Meta-Analysis. Front. Cardiovasc. Med..

