# Supplementary material for: A Meta-Analysis of Clinical and Echocardiographic Outcomes of Physiological Versus Conventional Pacing
Source: Biomedicines. 2025 May 31;13(6):1359. doi: 10.3390/biomedicines13061359 (PMC12189714; doi:10.3390/biomedicines13061359)
Supplement: Supplementary file 1 [file biomedicines-13-01359-s001.zip › biomedicines-3648333-supplementary.pdf]

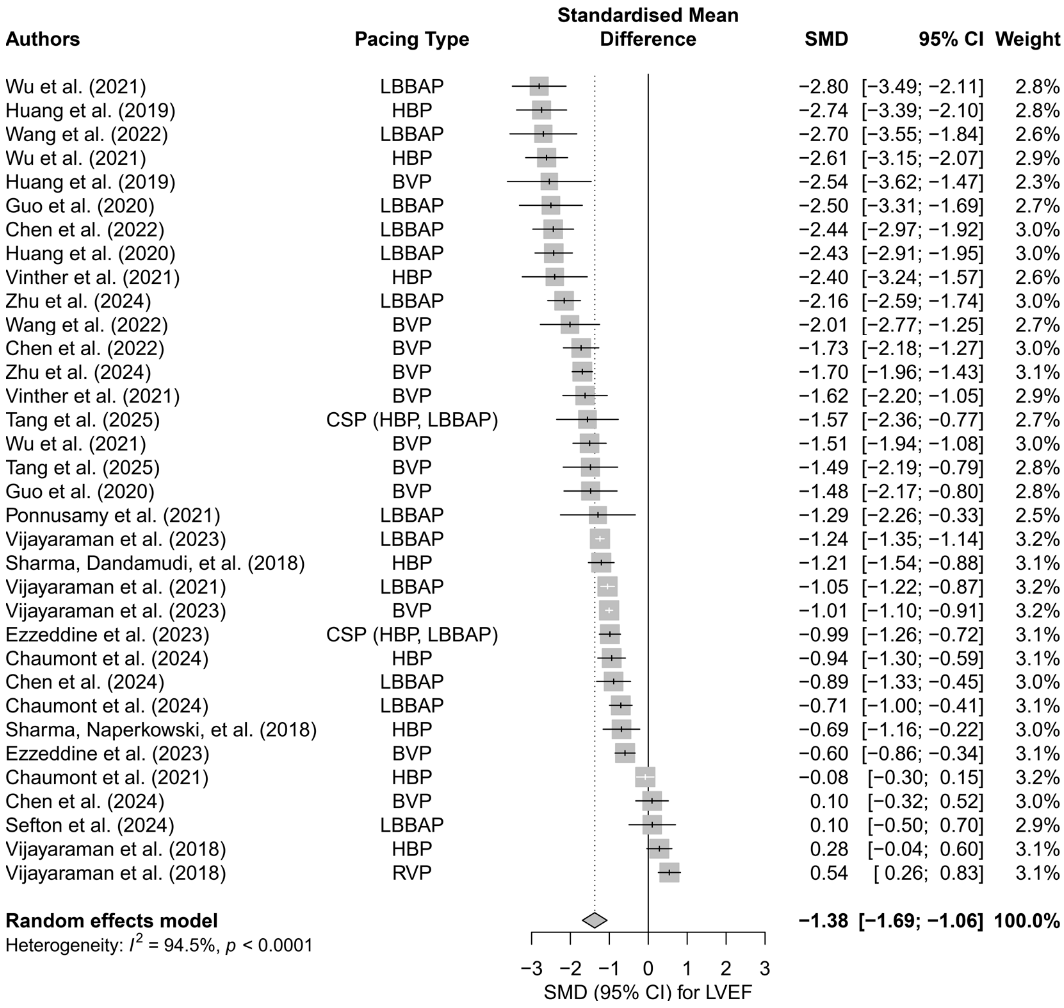

Figure S1. Forest plot for the overall meta-analysis of the effect of cardiac pacing on LVEF [21,30-32,34-39,41,43-45,48-51,53,55].

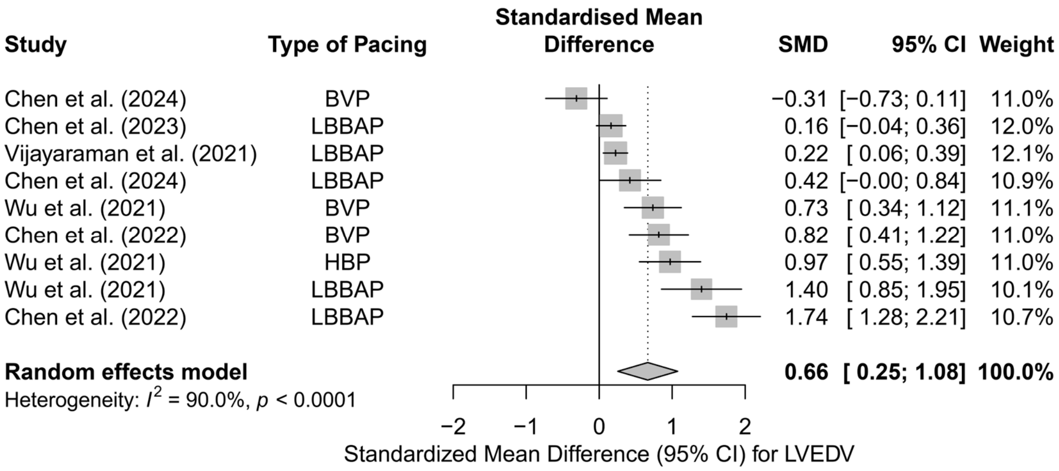

Figure S2. SMD in LVEDV after different types of cardiac pacing – random-effects model [32-34,48,53].

 https://doi.org/10.3390/biomedicines13061359

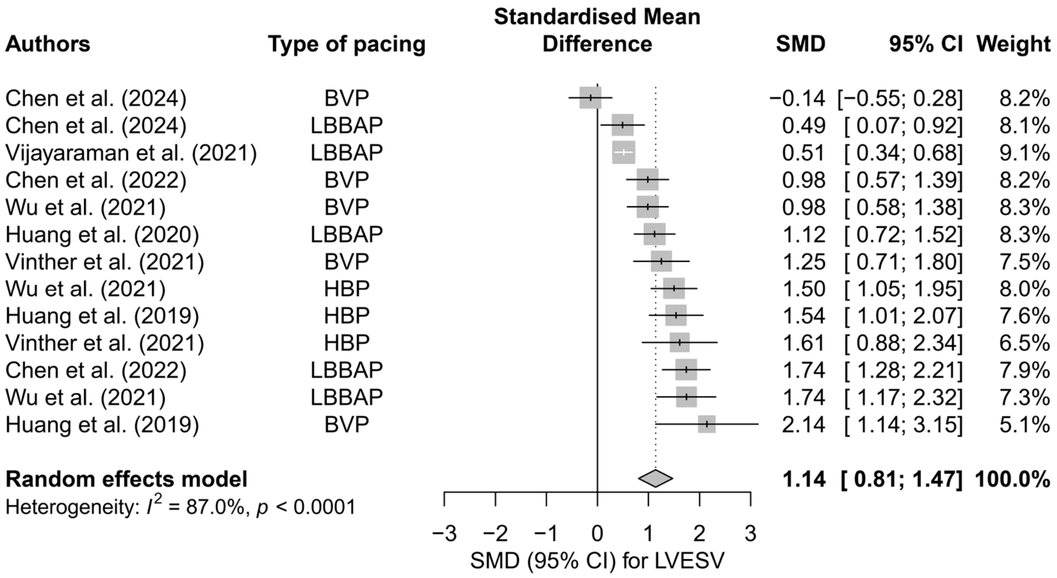

Figure S3. Forest plot of the overall meta-analysis of the effect of cardiac pacing (BVP, HBP, LBBAP) on the reduction of LVESV, expressed as SMD with 95%CI. Significant heterogeneity was observed (I^2 = 87.0%) [32,34,37,38,48,50,53].

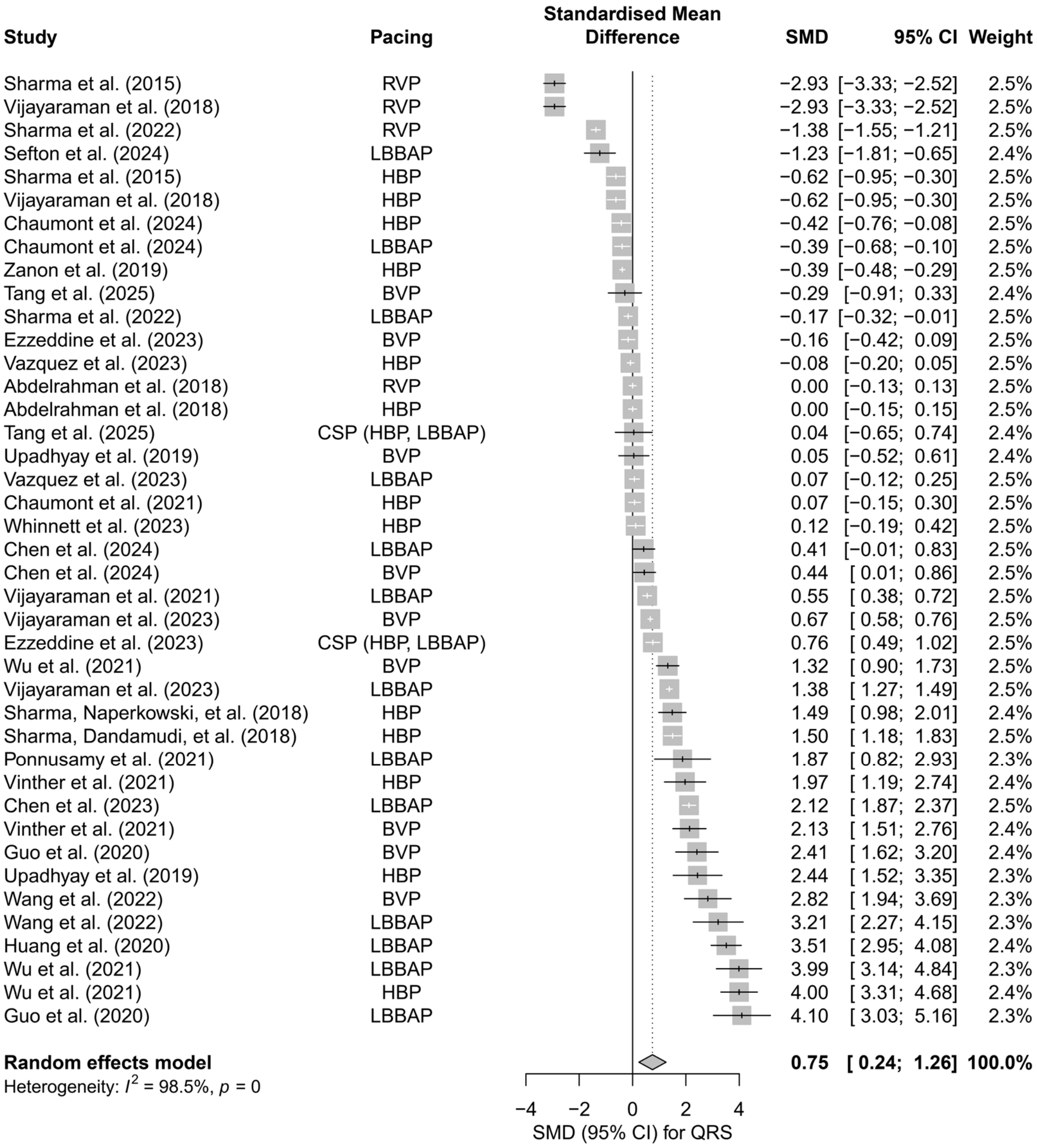

Figure S4. The overall effect of cardiac pacing on QRS duration. The forest plot presents the results of the meta-analysis of the SMD for changes in QRS duration across 41 comparisons from 24 publications, covering various pacing techniques. Positive SMD values indicate QRS shortening in the intervention group, while negative values indicate QRS prolongation. The pooled effect of the meta-analysis, calculated using a random-effects model (REML), was SMD = 0.75 (95%CI: 0.24–1.26; p = 0.0042), with very high heterogeneity (I^2 = 98.5%). Note: the p-value for heterogeneity (p = 0) was rounded by the RStudio environment; the actual p-value is less than 0.0001 [15,21,30,31,33-36,38-53].

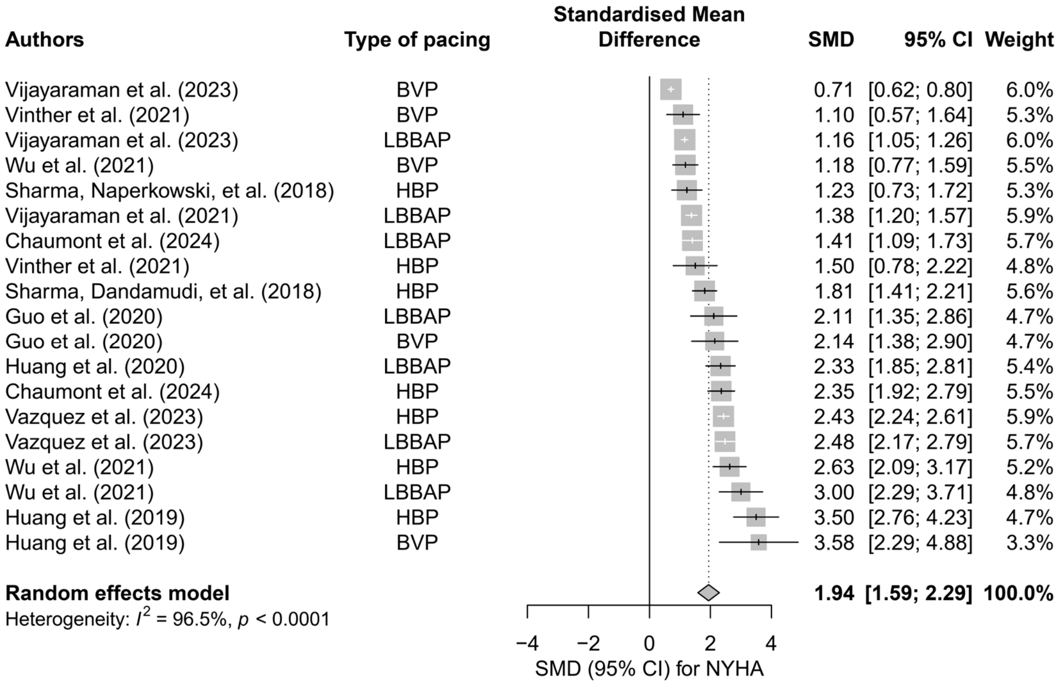

Figure S5. Cumulative meta-analysis of the effect of cardiac pacing on NYHA class. The forest plot presents the pooled effect of 19 comparisons assessing changes in NYHA class after implantation of HBP, LBBAP, and BVP systems. Positive SMD values indicate clinical improvement (a lower NYHA class after pacing). Note: Positive SMD values indicate improvement in NYHA (lower post-intervention class) [31,36-38,43,44,47-50,53].

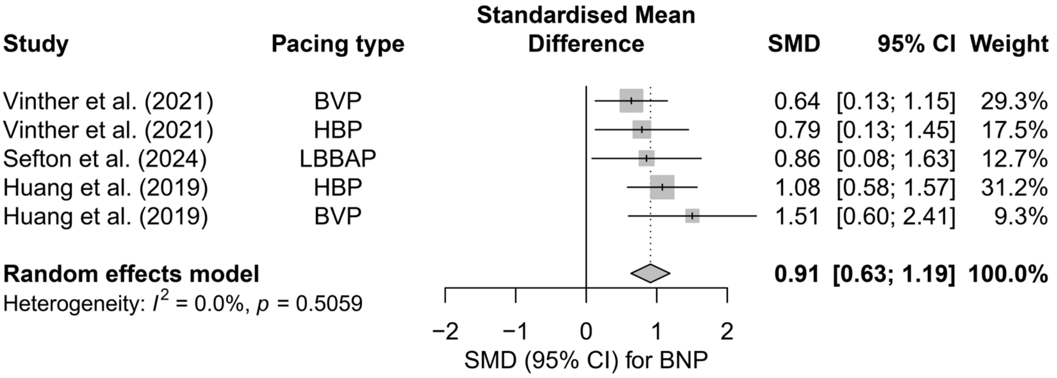

Figure S6. Cumulative forest plot illustrating the impact of different cardiac pacing techniques (BVP, HBP, LBBAP) on BNP levels. The forest plot presents the effect of various cardiac pacing techniques (BVP, HBP, LBBAP) on BNP levels, expressed as SMD with a 95%CI. All techniques were associated with a significant reduction in BNP [37,41,50].

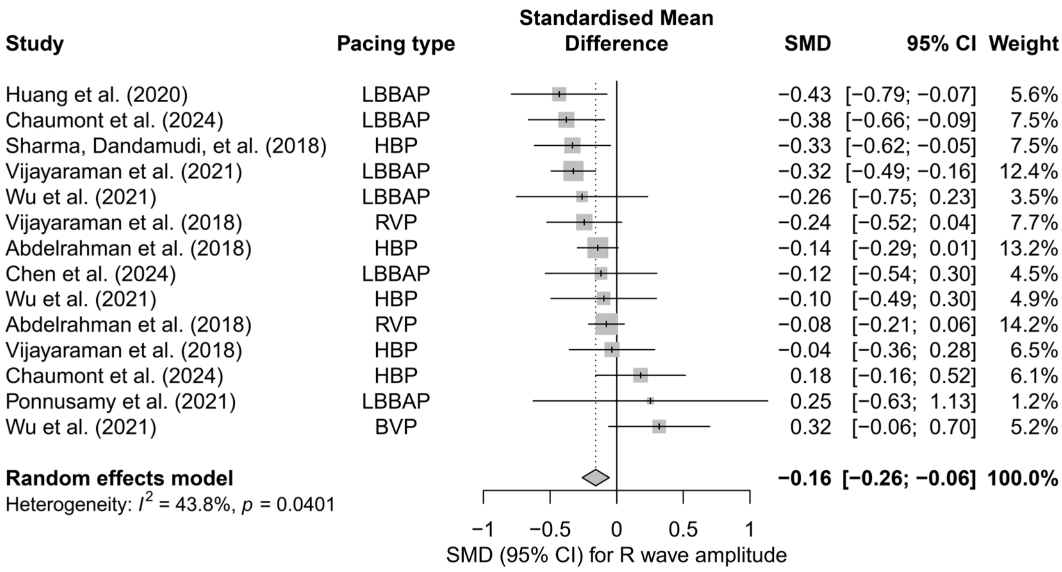

Figure S7. Forest plot presenting the results of the meta-analysis of changes in R-wave amplitude after the implantation of cardiac pacing systems, regardless of the pacing type. For each study arm, the standardized mean difference (SMD) between baseline and follow-up measurements is shown. Positive values indicate an increase in R-wave amplitude, while negative values indicate a decrease. The pooled effect of the meta-analysis indicates a statistically significant, small reduction in amplitude: SMD = –0.16 (95%CI: –0.26 to –0.06), p = 0.0024 [15,21,31,34,38,39,43,48,53].

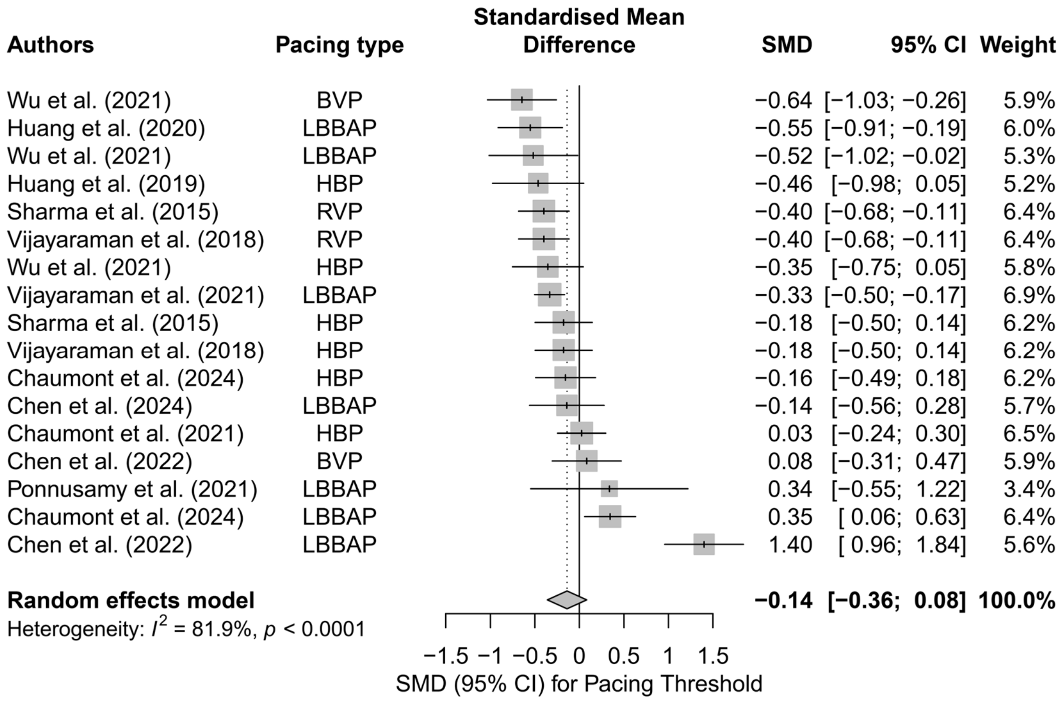

Figure S8. Forest plot presenting the standardized mean difference (SMD) in pacing threshold values between groups with different pacing techniques and control groups (k = 17). Negative values indicate a lower pacing threshold in the intervention group. The analysis, conducted using a random-effects model (REML), showed a non-significant overall effect (SMD = –0.14; 95%CI: –0.36 to 0.08; p = 0.22), with high heterogeneity (I^2 = 81.9%) [21,30-32,34,37-39,42,48,53].
